# Crystal structure and Hirshfeld surface analysis of (*E*)-*N*′-[4-(piperidin-1-yl)benzyl­idene]aryl­sulfono­hydrazides

**DOI:** 10.1107/S2056989018016237

**Published:** 2018-11-22

**Authors:** Nikhila Pai, Sabine Foro, B. Thimme Gowda

**Affiliations:** a Department of Chemistry, Mangalore University, Mangalagangotri-574 199, Mangalore, India; b Institute of Materials Science, Darmstadt University of Technology, Alarich-Weiss-Str. 2, D-64287, Darmstadt, Germany; c Karnataka State Rural Development and Panchayat Raj University, Gadag-582 101, India

**Keywords:** crystal structure, Hirshfeld surface analysis, fingerprint plots, Schiff bases, (*E*)-*N*′-[4-(piperidin-1-yl)benzyl­idene]aryl­sulfono­hydrazides

## Abstract

The crystal structures and Hirshfeld surface analyses of three Schiff bases, namely (*E*)-*N*′-[4-(piperidin-1-yl)benzyl­idene]benzene­sulfono­hydrazide, C_18_H_21_N_3_O_2_S, (I), (*E*)-4-methyl-*N*′-[4-(piperidin-1-yl)benzyl­idene]benzene­sulfono­hydrazide, C_19_H_23_N_3_O_2_S, (II), and (*E*)-4-chloro-*N*′-[4-(piperidin-1-yl)benzyl­idene]benzene­sulfono­hydrazide, C_18_H_20_ClN_3_O_2_S, (III), derived from aryl­sulfono­hydrazides and 4-(piperidin-4-yl)benzaldehyde have been analysed to investigate the effect of substituents on the structural parameters. All three structures crystallize in monoclinic crystal systems, in the space groups *P*2_1_/*c* for (I) and (II), and *C*2/*c* for (III). Compound (III) contains two independent mol­ecules in the asymmetric unit and sixteen mol­ecules per unit cell, while (I) and (II) both have one and four mol­ecules, respectively, in their asymmetric units and unit cells. In all cases, the central part of the mol­ecule is twisted at the S atom. In the crystals, the mol­ecules are linked *via* N—H⋯O hydrogen bonds, forming chains. Two-dimensional fingerprint plots of various inter­atomic contacts show that the major contributions are from H⋯H inter­actions.

## Abstract 2

New Schiff bases containing piperidine and aryl­sulfono­hydrazide moieties have been synthesized, characterized and their crystal structures determined to study the effect of substituents on the structural parameters. Their crystal structures are stabilized by N—H⋯O, C—H⋯O and O—H⋯O inter­actions. Two-dimensional fingerprint plots show that the largest contributions come from H⋯H inter­actions.

## Chemical context   

Piperidine is very common in many natural and synthetic N-containing medicaments and is present in the basic skeleton of many pharmacologically active compounds (Sampath, 2017 ▸). Compounds with a piperidine functional group are inter­mediates in the synthesis of various alkaloids (Wang & Wuorola, 1992 ▸; Grishina *et al.*, 1995 ▸). They are reported to be cholesterol-lowering (Comins *et al.*, 2001 ▸) and to display anti­viral (Kang *et al.*, 2015 ▸), anti-inflammatory, anti­oxidant (Tharini & Sangeetha, 2015 ▸), anti-epileptic (Kiasalari *et al.*, 2014 ▸), anti­microbial, anti­tumor and anti­fungal (Sahu *et al.*, 1979 ▸; Shah *et al.*, 1992 ▸) activities. Furthermore, Schiff bases find applications in the pharmacological field and are important in designing medicines (Parekh *et al.*, 2005 ▸). Thus the crystal structures of Schiff bases and piperidine derivatives have always been inter­esting, especially with regard to the stereochemistry across C=N and the conformation of the six-membered heterocyclic ring. We were inter­ested in exploring the effect of the substituents on the structural parameters of compounds containing these moieties. Thus we report herein the synthesis, characterization and crystal structures of (*E*)-*N*′-[4-(piperidin-1-yl)benzyl­idene]benzene­sulfono­hydrazide, C_18_H_21_N_3_O_2_S, (I), and its 4-methyl- and 4-chloro-derivatives, namely, (*E*)-4-methyl-*N*′-[4-(piperidin-1-yl)benzyl­idene]benz­ene­sulfono­hydrazide, C_19_H_23_N_3_O_2_S, (II), and (*E*)-4-chloro-*N*′-[4-(piperidin-1-yl)benzyl­idene]benzene­sulfono­hydrazide, C_18_H_20_ClN_3_O_2_S, (III).
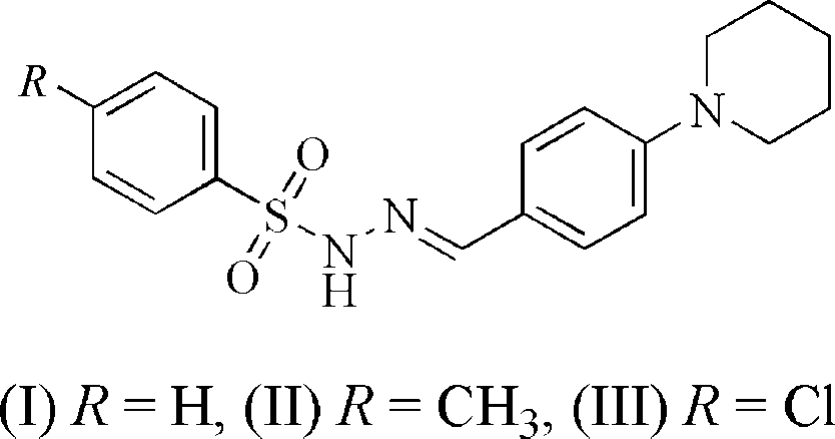


## Structural commentary   

**Figure 1:**
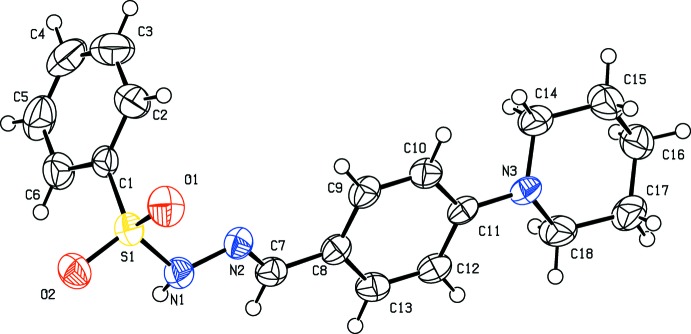
Mol­ecular structure of (I), showing the atom labelling and displacement ellipsoids drawn at the 50% probability level.

All three of the title compounds (Figs. 1 ▸–3 ▸ ▸) crystallize in the monoclinic crystal system but in space group *P*2_1_/*c* for (I) and (II), and space group *C*2/*c* for (III). The asymmetric units of compounds (I) and (II) each contain one mol­ecule whereas there are two independent mol­ecules in the asymmetric unit of (III). All the three compounds display an *E*-configuration about the C=N bond (Purandara *et al.*, 2017 ▸; Gu *et al.*, 2012 ▸), and a chair conformation of the piperidine ring.

In compounds (I) and (II) (Figs. 1 ▸ and 2 ▸), the sulfonamide bonds are found to be synclinal and the torsion angles of the sulfonamide moieties are −66.0 (2) and 63.5 (2)°, respectively (Moss, 1996 ▸). The dihedral angles between the phenyl ring (C1–C6/S1) and the mean plane of the N1/N2/C7–C9 hydrazone fragment are 85.3 (1) and 80.5 (1)° in (I) and (II), respectively, indicating that the hydrazone portion of the mol­ecules (C=N—N—S—C group) is not coplanar with the sulfonyl phenyl ring. The C7=N2 bond lengths of 1.271 (3) Å in (I) and 1.269 (3) Å in (II) are in agreement with double-bond character. In both compounds, the piperidine group is not sterically hindered. Thus the six-membered heterocyclic ring adopts the most stable chair conformation. The total puckering amplitude is 0.531 (3) Å in (I) and 0.465 (4) Å in (II), the puckering parameters are 173.7 (3), and 8.0 (5)° in (I) and (II), respectively, and the phase angles are 13.0 (3) in (I) and 184.0 (4)° in (II), respectively (Cremer & Pople, 1975 ▸; Nardelli, 1983 ▸). The C15—C14—N3—C11 torsion angles of −172.2 (2)° and 175.2 (3)° in (I) and (II), respectively, signify that the phenyl ring at the N atom of the piperidine ring is in an equatorial position (Nallini *et al.*, 2003 ▸).

The asymmetric unit of (III) contains two independent mol­ecules and the unit cell contains 16 mol­ecules. The torsion angles for the sulfonamide moieties in the two mol­ecules [C1—S1–N1–N2 = 59.7 (4)° and C19—S2—N4—N5 = 67.9 (4)°] signify a synclinal conformation (Moss, 1996 ▸). The hydrazone moiety (C=N—N—S—C group) and aryl­sulfonyl ring are not coplanar, with dihedral angles between the two planes of 87.3 (1) and 79.4 (1)°, respectively, in the first and second mol­ecules. The C7=N2 and C25=N5 bond lengths of 1.272 (5) and 1.269 (5) Å, respectively, are consistent with double-bond character. As in compounds (I) and (II), the piperidine group in (III) adopts a chair conformation, with the total puckering amplitude of *Q_T_* = 0.283 (7) and 0.475 (1) Å in the first and second mol­ecules, respectively, *θ* = 2.7 (14), 175.5 (8)° and phase angles *φ* = 220 (22)° and 353 (10)° in the two mol­ecules, respectively. The phenyl ring at the piperidine N atom is equatorial, as is evident from C15—C14—N3—C11 and C33—C32—N6—C29 torsion angles of 174.4 (7) and −168.9 (5)°, respectively.

## Supra­molecular features   

**Figure 4:**
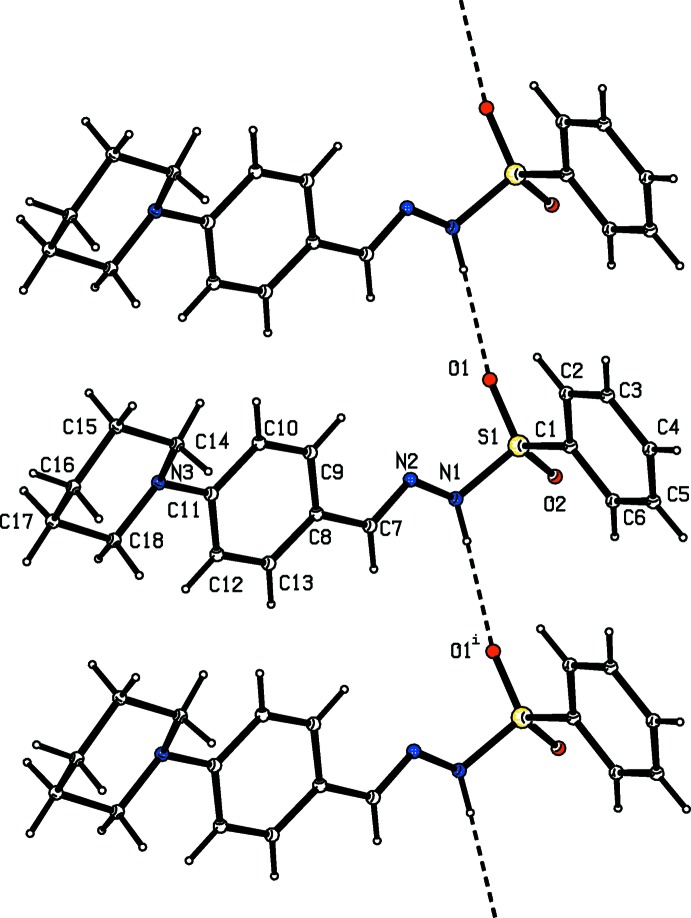
Hydrogen-bonding pattern in (I) with hydrogen bonds shown as dashed lines. Symmetry code as in Table 1 ▸.

**Figure 5:**
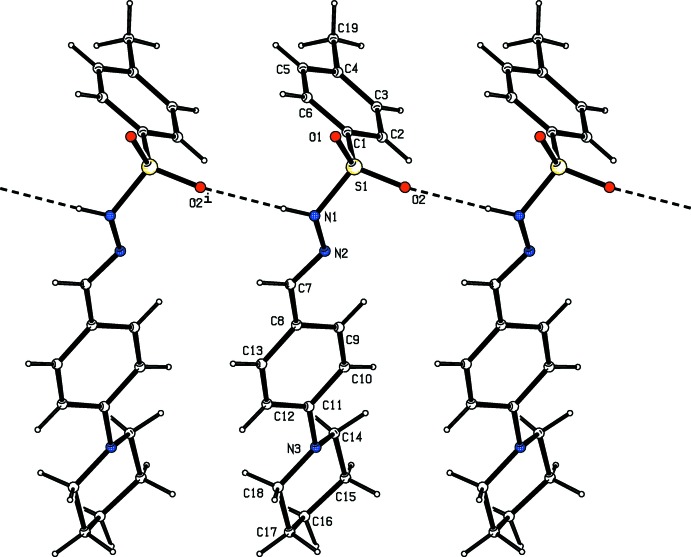
Hydrogen-bonding pattern in (II) with hydrogen bonds shown as dashed lines. Symmetry code as in Table 2 ▸.

**Figure 6:**
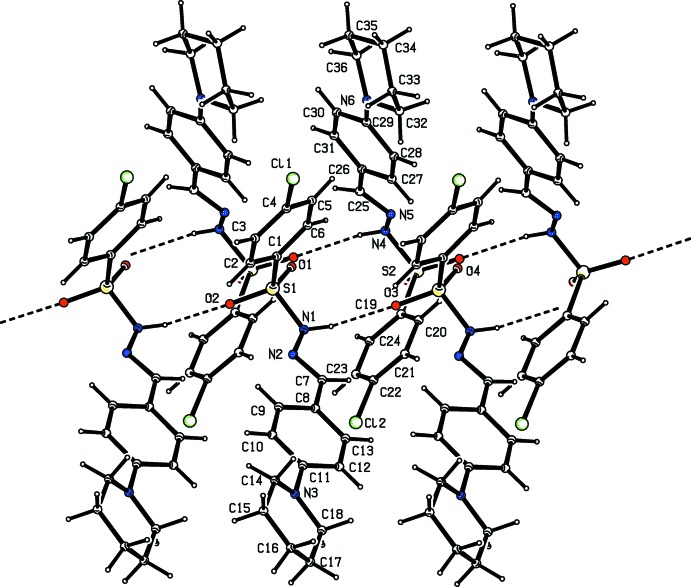
Hydrogen-bonding pattern in (III) with hydrogen bonds shown as dashed lines.

**Figure 7:**
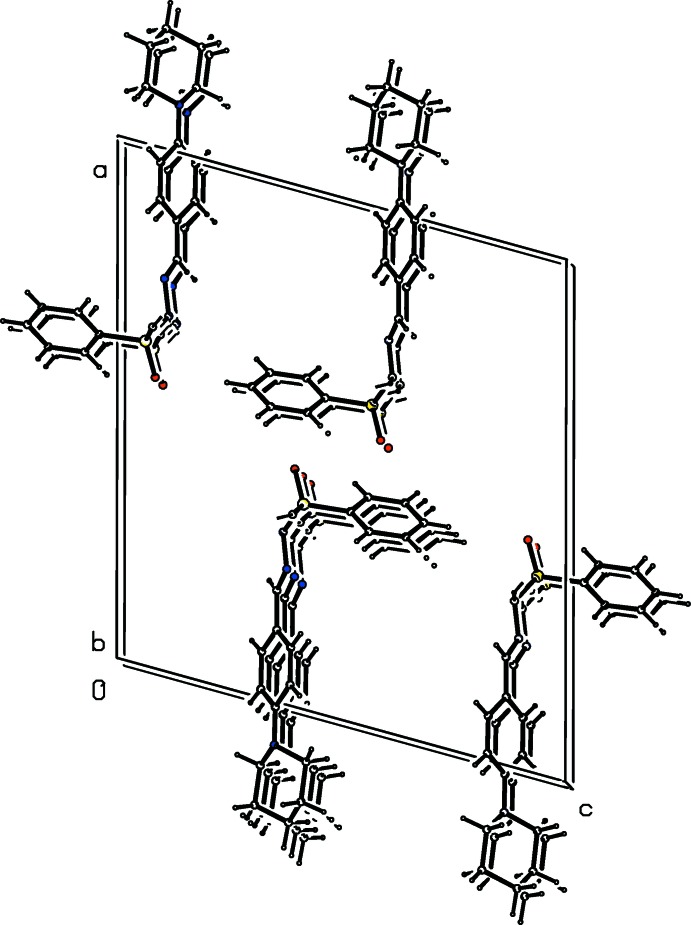
Mol­ecular packing of (I).

**Figure 8:**
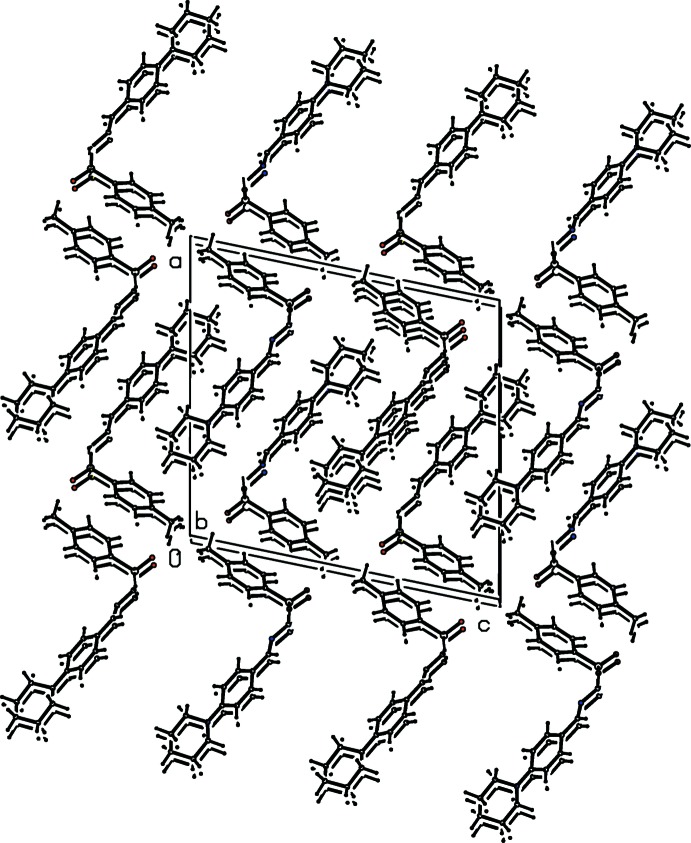
Mol­ecular packing of (II).

**Figure 9:**
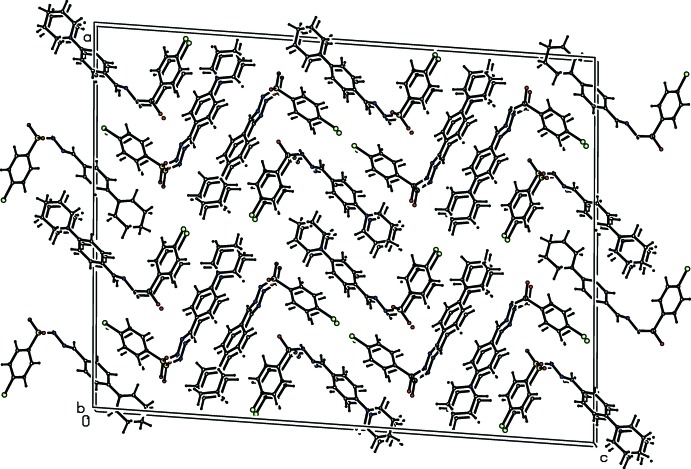
Mol­ecular packing of (III).

In all the three crystal structures, the amino H atom of the sulfono­hydrazide segment acts as a donor and the sulfonyl O atom acts as an acceptor in N—H⋯O hydrogen-bonding inter­actions that generate *C*4 chains propagating parallel to the *b* axis (Tables 1 ▸–3 ▸ ▸, Figs. 4–9 ▸ ▸ ▸ ▸ ▸ ▸). Substitution at the *para* position by a methyl or chloro group to produce compounds (II) and (III) has no remarkable effect on the hydrogen-bonding pattern.

## Database survey   

Although there are several reports on the crystal structures of piperidine or sulfonyl­hydrazides derivatives, reports on the crystal structures of 4-(piperidin-1-yl)benzaldehyde functionalized with sulfonyl­hydrazides are very few. Comparison of the present data with those of thio­phene/phenyl-piperidine hybrid chalcones (Parvez *et al.*, 2014 ▸) reveals that the compounds also adopt *E* configuration around the C=N bond and the piperidine rings exhibit a chair conformation. A chair conformation of the piperidine ring is also found in 5-nitro-2-(piperidin-1-yl)benzaldehyde (N’Gouan *et al.*, 2009 ▸) and (5-nitro-2-piperidino)­benzyl­idene *p*-toluene­sulfonyl­hydra­zone (Yapo *et al.*, 2008 ▸).

## Hirshfeld surface analysis   

**Figure 10:**
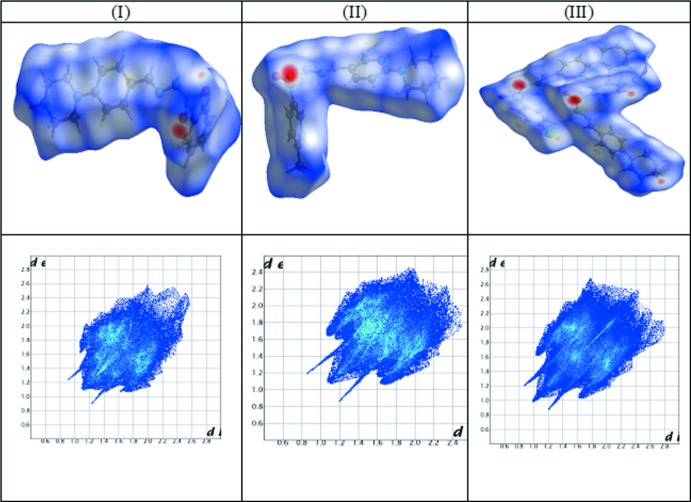
Top: Hirshfeld surface mapped over *d*_norm_ for (I), (II) and (III). Bottom: two-dimensional fingerprint plots for (I), (II) and (III).

Hirshfeld surfaces (HS) and 2D fingerprint plots were generated using *CrystalExplorer17* (Turner *et al.*, 2017 ▸; McKinnon *et al.*, 2007 ▸; Spackman & Jayatilaka, 2009 ▸). The terms such as *d*_norm_, *d*_i_ and *d*_e_ are defined in the usual way (Shit *et al.*, 2016 ▸). The function *d*_norm_ is a ratio enclosing the distances of any surface point to the nearest inter­ior (*d*_i_) and exterior (*d*_e_) atom and the van der Waals radii of the atoms (Hirshfeld, 1977 ▸; Soman *et al.*, 2014 ▸). The function *d*_norm_ will be equal to zero when inter­molecular distances are close to van der Waals contacts. They are indicated by a white colour on the HS, while contacts longer than the sum of van der Waals radii with positive *d*_norm_ values are coloured in blue. The surface images and plots for *d*_norm_ (Fig. 10 ▸) were generated using a high standard surface resolution over a colour scale of −0.3495 to 1.3559, −0.4124 to 1.6768 and −0.3876 to 1.5649 a.u. for (I), (II) and (III), respectively.

**Figure 11:**
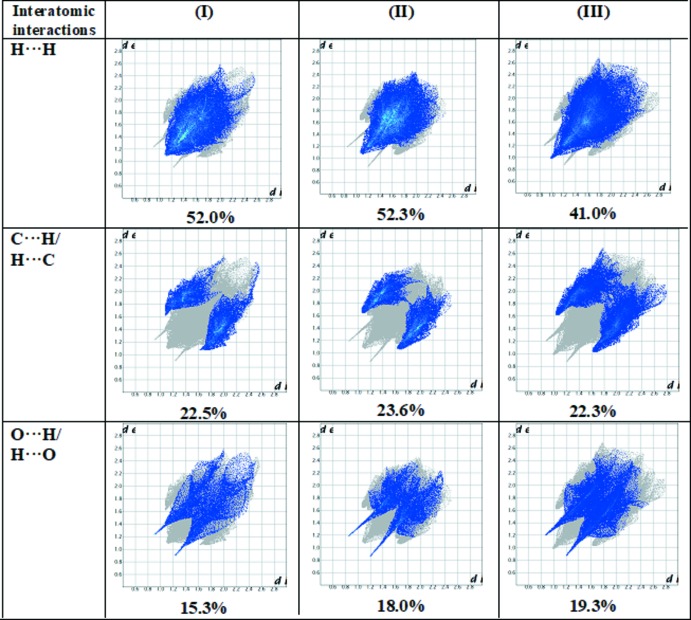
Two-dimensional fingerprint plots for (I), (II) and (III), showing the contributions of the different types of inter­actions.

Hirshfeld fingerprint plots for various inter­actions show differences in the percentage contributions to the Hirshfeld surfaces. H⋯H contacts make the maximum contribution to the Hirshfeld surfaces in all three compounds. The contributions of significant contacts in the three compounds are in the following order: H⋯H, C⋯H/H⋯C and O⋯H/H⋯O. In compound (I), these inter­actions cover a region of 52.0% (*d*_i_ = *d*_e_ = 1.5 Å), 22.5% (*d*_i_ + *d*_e_ = 3.2 Å), and 15.3% (*d*_i_ + *d*_e_ = 2.4 Å) (Fig. 11 ▸), respectively. The other inter­atomic contacts and percentages of contributions to the Hirshfeld surface are N⋯H/H⋯N (6.7%), C⋯O/O⋯C (3.1%). In compound (II), the contributions of the various contacts are: H⋯H 52.3% (*d*_i_ = *d*_e_ =1.5 Å), C⋯H/H⋯C 23.6% (*d*_i_ + *d*_e_ = 3.2 Å), and O⋯H/H⋯O 18.0% (*d*_i_ + *d*_e_ = 2.4 Å) (Fig. 11 ▸). Among the minor contributions observed, N⋯H/H⋯N inter­action cover a region of 6.1%. In the case of compound (III), the major contributions are H⋯H 41.0% (*d*_i_ = *d*_e_ = 1.0 Å), C⋯H/H⋯C 22.3% (3.2 Å) and O⋯H/H⋯O, 19.3% (*d*_i_ + *d*_e_ = 2.4 Å) along with minor contributions from Cl⋯H/H⋯Cl (9.5%) and N⋯H/H⋯N (5.1%) inter­actions (Fig. 11 ▸).

## Synthesis and crystallization   

**Synthesis of benzene­sulfono­hydrazide and 4-methyl and 4-chloro-benzene­sulfono­hydrazides**

To solutions of hydrazine hydrate (99%) (0.03mol) in THF at 273 K under stirring, a solution of benzene­sulfonyl chloride, 4-methyl­benzene­sulfonyl chloride or 4-chloro­benzene­sulfonyl chloride (0.02 mol) in THF was added dropwise. Three separ­ate reaction mixtures were kept under stirring at 273 K for 1 h and stirring continued for 24 h at room temperature. The formation of the products was monitored by TLC. After completion of the reactions, the reaction mixtures were poured separately onto ice-cold water. The separated solids, benzene­sulfono­hydrazide, 4-methyl­benzene­sulfono­hydrazide or 4-chloro­benzene­sulfono­hydrazide, were filtered off and dried. The products were recrystallized from ethanol solution to get the pure products.

The purity of the compounds was checked by TLC and they were characterized by their IR spectra. They were further characterized by ^1^H and ^13^C NMR spectra. The characteristic IR absorptions and ^1^H and ^13^C NMR signals are as follows:

**Benzene­sulfono­hydrazide:** m.p. 374–376 K; FT–IR (ATR, ν_max_, cm^−1^): 3254.4 (*s*, NH_2_ str), 3198.3 (*s*, N—H str), 1325.1 (*s*, S=O asym str) and 1140.8 (*vs*, S=O sym str).

^1^H and ^13^C NMR spectra: ^1^H (400 MHz, DMSO-*d*_6_, δ, ppm): 7.93–7.42 (*m*, 5H, Ar—H), 5.85 (*t*, 1H), 3.43 (*d*, 2H). ^13^C NMR (100 MHz, DMSO-*d*_6_, δ, ppm); 134.57, 130.15, 129.12, 125.63.

**4-Methyl­benzene­sulfono­hydrazide:** m.p. 382–385 K; FT–IR (ATR, ν_max_, cm^−1^): 3245.1 (*s*, NH_2_ str), 3193.8 (*s*, N—H str), 1330.5 (*s*, S=O asym str) and 1126.5 (*vs*, S=O sym str).

^1^H and ^13^C NMR spectra: ^1^H (400 MHz, DMSO-*d*_6_, δ, ppm); 7.71–7.31 (*m*, 4H, Ar—H), 5.91 (*t*, 1H), 3.48 (*d*, 2H), 2.19 (*s*, 3H, CH_3_). ^13^C NMR (100 MHz, DMSO-*d*_6_, δ, ppm); 142.36, 136.90, 128.13, 126.71, 22.11.

**4-Chloro­benzene­sulfono­hydrazide:** m.p. 388–90 K; FT–IR (ATR, ν_max_, cm^−1^): 3259.4 (*s*, NH_2_ str), 3195.1 (*s*, N—H str), 1341.7 (*s*, S=O asym str) and 1138.5 (*vs*, S=O sym str).

^1^H and ^13^C NMR spectra: ^1^H (400 MHz, DMSO-*d*_6_, δ, ppm); 7.58–7.67 (*m*, 5H, Ar—H), 5.87 (*t*, 1H), 3.41 (*d*, 2H). ^13^C NMR (100 MHz, DMSO-d_6_, δ, ppm); 137.90, 137.29, 130.30, 128.42.

**Synthesis of the title compounds (I), (II) and (III):**

Mixtures of 4-(piperidin-1-yl)benzaldehyde (0.001 mol) and benzene­sulfono­hydrazide, 4-methyl­benzene­sulfono­hydrazide or 4-chloro­benzene­sulfono­hydrazide (0.001 mol) in ethanol (10 ml) and two drops of glacial acetic acid were stirred at room temperature for 2 h. The formation of the products was monitored by TLC. The reaction mixtures were separately poured on crushed ice and the solids that formed were washed and dried. The products were recrystallized to constant melting points from an aceto­nitrile:DMF (5:1 *v*:*v*) mixture. The purity of the compounds was checked by TLC and they were characterized by their IR spectra. They were further characterized by ^1^H and ^13^C NMR spectra. The characteristic IR absorptions and ^1^H and ^13^C NMR signals are as follows

**Compound (I)**: m.p. 417–419 K; FT–IR (ATR, ν_max_, cm^−1^): 3219.2 (*s*, N—H str), 1609.3 (*s*, C=N str), 1363.7 (*s*, S=O asym str) and 1165.0 (*vs*, S=O sym str).

^1^H and ^13^C NMR spectra: ^1^H (400 MHz, DMSO-*d*_6_, δ, ppm); 9.41 (*s*, 1H, N—H), 8.39 (*s*, 1H, =C—H), 7.76–7.59 (*m*, 5H, Ar—H), 7.54–6.54 (*m*, 4H, Ar—H), 3.46–1.82 (*m*, 4H), 1.47–1.39 (*m*, 6H). ^13^C NMR (100 MHz, DMSO-*d*_6_, δ, ppm); 151.34, 147.31, 138.89, 133.62, 130.94, 129.91, 128.27, 124.97, 112.76, 48.54, 24.82, 23.93.

**Compound (II):** m.p. 439 − 441 K; FT–IR (ATR, ν_max_, cm^−1^): 3214.3 (*s*, N—H, str), 1606.7 (*s*, C=N str), 1359.82 (*s*, S=O asym) and 1163.08cm^−1^ (*vs*, S=O sym).

^1^H and ^13^C spectra: ^1^H (400 MHz, DMSO-*d*_6_, δ, ppm); 10.98 (*s*, 1H, N—H), 7.77–7.75 (*m*, 3H, Ar—H, =C—H), 7.37–7.34 (*m*, 4H, Ar—H), 3.29–2.36 (*m*, 4H), 2.37 (*s*, 3H, CH_3_), 1.56–1.47 (*m*, 6H); ^13^C NMR (100 MHz, DMSO-*d*_6_ δ, ppm); 152.28, 147.48, 142.92, 136.34, 129.28, 127.89, 127.17, 114.45, 48.51, 24.92, 23.87, 21.0.

**Compound (III):** m.p. 429–431 K; FT–IR (ATR, ν_max_, cm^−1^): 3213.4 (*s*, N—H, str), 1608.9 (*s*, C=N, str), 1365.6 (*s*, S=O asym str) and 1166.9 (*vs*, S=O sym str).

^1^H and ^13^C spectra: ^1^H (400 MHz, DMSO-*d*_6_, δ, ppm); 8.18 (*s*, 1H, N-H), 7.91–7.88 (*m*, 2H, Ar—H), 7.70 (*s*, 1H, =C—H), 7.45–7.40 (*m*, 4H), 6.82 (*d*, 2H, Ar—H), 3.26–3.23 (*m*, 4H), 1.69–1.60 (*m*, 6H). ^13^C NMR (100 MHz, DMSO-*d*_6_, δ, ppm); 153.13, 150.03, 139.68, 136.87, 129.41, 129.27, 128.88, 122.65, 114.85, 49.29, 25.41, 24.28.

Prismatic single crystals of the compounds used in X-ray diffraction studies were grown from their solutions in a aceto­nitrile–DMF (5:1 *v*:*v*) mixture by slow evaporation of the solvent.

## Refinement   

Crystal data, data collection and structure refinement details are summarized in Table 4 ▸. H atoms bonded to C were positioned with idealized geometry and refined using a riding model with the aromatic C—H = 0.93, 0.96 (meth­yl), or 0.97 Å (methyl­ene). H atoms of the NH groups were located in a difference map and their positions refined. All H atoms were refined with *U*_iso_(H) = 1.2*U*_eq_(C-aromatic, C-methyl­ene, N) or 1.5*U*_eq_(C-meth­yl). In compound (III), the *U* ^ij^ components of atoms C14, C15, C17, and C18 were restrained to approximate isotropic behaviour.

## Supplementary Material

Crystal structure: contains datablock(s) I, II, III, global. DOI: 10.1107/S2056989018016237/tx2009sup1.cif

Structure factors: contains datablock(s) I. DOI: 10.1107/S2056989018016237/tx2009Isup2.hkl

Structure factors: contains datablock(s) II. DOI: 10.1107/S2056989018016237/tx2009IIsup3.hkl

Structure factors: contains datablock(s) III. DOI: 10.1107/S2056989018016237/tx2009IIIsup4.hkl

CCDC references: 1879247, 1879246, 1879245

Additional supporting information:  crystallographic information; 3D view; checkCIF report

## Figures and Tables

**Figure 2:**
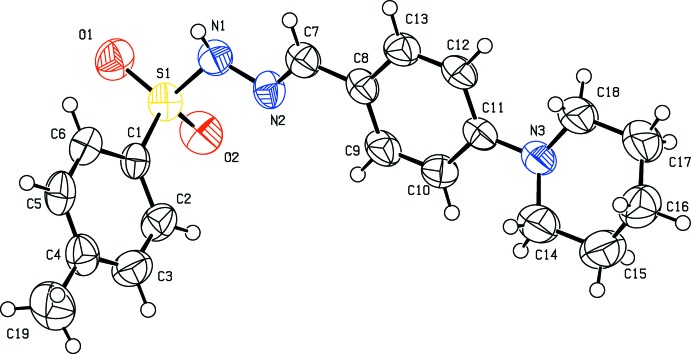
Mol­ecular structure of (II), showing the atom labelling and displacement ellipsoids drawn at the 50% probability level.

**Figure 3:**
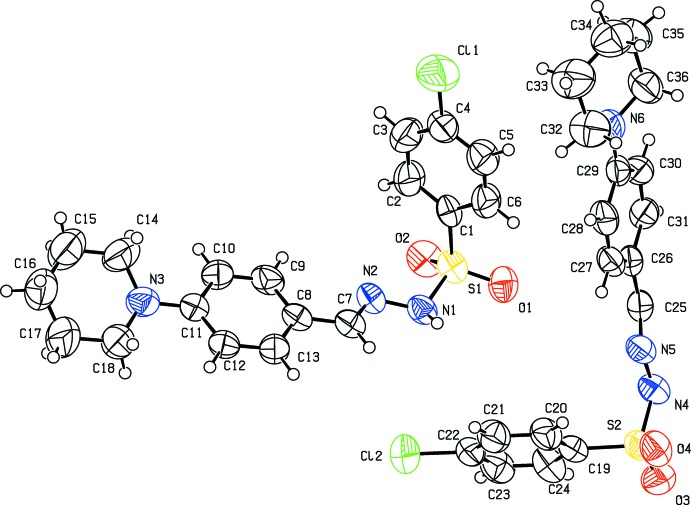
Mol­ecular structure of (III), showing the atom labelling and displacement ellipsoids drawn at the 50% probability level.

**Table 1:** Hydrogen-bond geometry (Å, °) for (I)

*D*—H⋯*A*	*D*—H	H⋯*A*	*D*⋯*A*	*D*—H⋯*A*
N1—H1*N*⋯O1^i^	0.84 (2)	2.32 (2)	3.133 (3)	165 (2)

Symmetry code: (i) 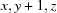.

**Table 2:** Hydrogen-bond geometry (Å, °) for (II)

*D*—H⋯*A*	*D*—H	H⋯*A*	*D*⋯*A*	*D*—H⋯*A*
N1—H1*N*⋯O2^i^	0.79 (3)	2.29 (3)	3.068 (3)	170 (3)

Symmetry code: (i) 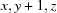.

**Table 3:** Hydrogen-bond geometry (Å, °) for (III)

*D*—H⋯*A*	*D*—H	H⋯*A*	*D*⋯*A*	*D*—H⋯*A*
N1—H1*N*⋯O2^i^	0.83 (4)	2.26 (5)	3.025 (5)	153 (5)
N4—H4*N*⋯O4^ii^	0.84 (5)	2.29 (5)	3.115 (6)	169 (5)

Symmetry codes: (i) 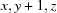; (ii) 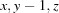.

**Table 4:** Experimental details

	(I)	(II)	(III)
Crystal data			
Chemical formula	C_18_H_21_N_3_O_2_S	C_19_H_23_N_3_O_2_S	C_18_H_20_ClN_3_O_2_S
*M*_r_	343.44	357.46	377.88
Crystal system, space group	Monoclinic, *P*2_1_/*c*	Monoclinic, *P*2_1_/*c*	Monoclinic, *C*2/*c*
Temperature (K)	293	293	293
*a*, *b*, *c* (Å)	19.221 (2), 5.4270 (7), 17.143 (2)	18.442 (2), 5.3250 (4), 19.412 (2)	33.052 (6), 5.258 (1), 43.026 (8)
β (°)	105.45 (2)	101.74 (1)	94.05 (2)
*V* (Å^3^)	1723.6 (4)	1866.5 (3)	7459 (2)
*Z*	4	4	16
Radiation type	Mo *K*α	Mo *K*α	Mo *K*α
μ (mm^−1^)	0.20	0.19	0.33
Crystal size (mm)	0.44 × 0.32 × 0.28	0.48 × 0.24 × 0.10	0.50 × 0.26 × 0.14

Data collection			
Diffractometer	Oxford Diffraction Xcalibur diffractometer with Sapphire CCD	Oxford Diffraction Xcalibur diffractometer with Sapphire CCD	Oxford Diffraction Xcalibur diffractometer with Sapphire CCD
Absorption correction	Multi-scan (*CrysAlis RED*; Oxford Diffraction, 2009 ▸)	Multi-scan (*CrysAlis RED*; Oxford Diffraction, 2009 ▸)	Multi-scan (*CrysAlis RED*; Oxford Diffraction, 2009 ▸)
*T*_min_, *T*_max_	0.916, 0.945	0.914, 0.981	0.851, 0.955
No. of measured, independent and observed [*I* > 2σ(*I*)] reflections	6052, 3164, 2475	6735, 3422, 2166	14002, 6833, 2999
*R*_int_	0.019	0.026	0.042
(sin θ/λ)_max_ (Å^−1^)	0.602	0.602	0.602

Refinement			
*R*[*F* ^2^ > 2σ(*F* ^2^)], *wR*(*F* ^2^), *S*	0.043, 0.108, 1.06	0.051, 0.137, 1.02	0.075, 0.172, 1.01
No. of reflections	3164	3422	6833
No. of parameters	220	230	457
No. of restraints	0	0	31
H-atom treatment	H atoms treated by a mixture of independent and constrained refinement	H atoms treated by a mixture of independent and constrained refinement	H atoms treated by a mixture of independent and constrained refinement
Δρ_max_, Δρ_min_ (e Å^−3^)	0.24, −0.31	0.21, −0.22	0.27, −0.30

Computer programs: *CrysAlis CCD* and *CrysAlis RED* (Oxford Diffraction, 2009 ▸), *SHELXS2013/1* (Sheldrick, 2008 ▸), *SHELXL2014/6* (Sheldrick, 2015 ▸) and *PLATON* (Spek, 2003 ▸).

## supplementary crystallographic information

### (E)-N'-[4-(Piperidin-1-yl)benzylidene]benzenesulfonohydrazide (I) Crystal data

**Table:**

C_18_H_21_N_3_O_2_S	*F*(000) = 728
*M**_r_* = 343.44	*D*_x_ = 1.323 Mg m^−^^3^
Monoclinic, *P*2_1_/*c*	Mo *K*α radiation, λ = 0.71073 Å
*a* = 19.221 (2) Å	Cell parameters from 2039 reflections
*b* = 5.4270 (7) Å	θ = 2.8–27.8°
*c* = 17.143 (2) Å	µ = 0.20 mm^−^^1^
β = 105.45 (2)°	*T* = 293 K
*V* = 1723.6 (4) Å^3^	Prism, colourless
*Z* = 4	0.44 × 0.32 × 0.28 mm

### (E)-N'-[4-(Piperidin-1-yl)benzylidene]benzenesulfonohydrazide (I) Data collection

**Table:**

Oxford Diffraction Xcalibur diffractometer with Sapphire CCD	2475 reflections with *I* > 2σ(*I*)
Radiation source: Enhance (Mo) X-ray Source	*R*_int_ = 0.019
Rotation method data acquisition using ω scans.	θ_max_ = 25.4°, θ_min_ = 2.8°
Absorption correction: multi-scan (CrysAlis RED; Oxford Diffraction, 2009)	*h* = −23→20
*T*_min_ = 0.916, *T*_max_ = 0.945	*k* = −4→6
6052 measured reflections	*l* = −19→20
3164 independent reflections	

### (E)-N'-[4-(Piperidin-1-yl)benzylidene]benzenesulfonohydrazide (I) Refinement

**Table:**

Refinement on *F*^2^	0 restraints
Least-squares matrix: full	Hydrogen site location: mixed
*R*[*F*^2^ > 2σ(*F*^2^)] = 0.043	H atoms treated by a mixture of independent and constrained refinement
*wR*(*F*^2^) = 0.108	*w* = 1/[σ^2^(*F*_o_^2^) + (0.0438*P*)^2^ + 0.7956*P*] where *P* = (*F*_o_^2^ + 2*F*_c_^2^)/3
*S* = 1.06	(Δ/σ)_max_ = 0.001
3164 reflections	Δρ_max_ = 0.24 e Å^−^^3^
220 parameters	Δρ_min_ = −0.31 e Å^−^^3^

### (E)-N'-[4-(Piperidin-1-yl)benzylidene]benzenesulfonohydrazide (I) Special details

**Table:**

Geometry. All esds (except the esd in the dihedral angle between two l.s. planes) are estimated using the full covariance matrix. The cell esds are taken into account individually in the estimation of esds in distances, angles and torsion angles; correlations between esds in cell parameters are only used when they are defined by crystal symmetry. An approximate (isotropic) treatment of cell esds is used for estimating esds involving l.s. planes.

### (E)-N'-[4-(Piperidin-1-yl)benzylidene]benzenesulfonohydrazide (I) Fractional atomic coordinates and isotropic or equivalent isotropic displacement parameters (Å^2^)

**Table:**

	*x*	*y*	*z*	*U*_iso_*/*U*_eq_	
C1	0.60577 (10)	0.1520 (4)	0.46537 (12)	0.0384 (5)	
C2	0.63592 (13)	−0.0066 (5)	0.42141 (15)	0.0553 (6)	
H2	0.6625	−0.1422	0.4460	0.066*	
C3	0.62614 (16)	0.0387 (6)	0.33995 (17)	0.0720 (8)	
H3	0.6459	−0.0684	0.3093	0.086*	
C4	0.58782 (15)	0.2386 (6)	0.30389 (15)	0.0699 (8)	
H4	0.5821	0.2681	0.2491	0.084*	
C5	0.55771 (13)	0.3960 (6)	0.34822 (16)	0.0692 (8)	
H5	0.5311	0.5309	0.3232	0.083*	
C6	0.56663 (12)	0.3554 (5)	0.42987 (14)	0.0551 (6)	
H6	0.5467	0.4627	0.4603	0.066*	
C7	0.79265 (11)	0.4355 (4)	0.63390 (11)	0.0403 (5)	
H7	0.7761	0.5782	0.6531	0.048*	
C8	0.86830 (11)	0.4209 (4)	0.63351 (11)	0.0378 (5)	
C9	0.89690 (11)	0.2255 (4)	0.59921 (12)	0.0434 (5)	
H9	0.8667	0.0973	0.5749	0.052*	
C10	0.96855 (11)	0.2183 (4)	0.60049 (12)	0.0431 (5)	
H10	0.9857	0.0851	0.5770	0.052*	
C11	1.01676 (10)	0.4064 (4)	0.63636 (11)	0.0353 (4)	
C12	0.98802 (11)	0.5993 (4)	0.67192 (12)	0.0428 (5)	
H12	1.0181	0.7265	0.6972	0.051*	
C13	0.91592 (12)	0.6049 (4)	0.67031 (12)	0.0435 (5)	
H13	0.8987	0.7361	0.6947	0.052*	
C14	1.10674 (12)	0.2969 (5)	0.56571 (14)	0.0529 (6)	
H14A	1.0889	0.4113	0.5214	0.063*	
H14B	1.0816	0.1418	0.5511	0.063*	
C15	1.18625 (13)	0.2563 (5)	0.57619 (18)	0.0657 (7)	
H15A	1.2026	0.1212	0.6137	0.079*	
H15B	1.1941	0.2101	0.5245	0.079*	
C16	1.22997 (12)	0.4823 (5)	0.60752 (15)	0.0558 (6)	
H16A	1.2810	0.4462	0.6174	0.067*	
H16B	1.2178	0.6135	0.5678	0.067*	
C17	1.21388 (12)	0.5618 (5)	0.68471 (14)	0.0595 (7)	
H17A	1.2402	0.7120	0.7040	0.071*	
H17B	1.2302	0.4356	0.7256	0.071*	
C18	1.13426 (12)	0.6062 (5)	0.67295 (15)	0.0573 (6)	
H18A	1.1257	0.6457	0.7248	0.069*	
H18B	1.1195	0.7471	0.6376	0.069*	
N1	0.67909 (9)	0.2983 (4)	0.61782 (11)	0.0422 (4)	
H1N	0.6650 (12)	0.444 (4)	0.6160 (14)	0.051*	
N2	0.74846 (9)	0.2602 (3)	0.60892 (10)	0.0412 (4)	
N3	1.09002 (9)	0.3935 (3)	0.63815 (9)	0.0387 (4)	
O1	0.64512 (9)	−0.1391 (3)	0.58934 (10)	0.0571 (4)	
O2	0.55362 (8)	0.1803 (3)	0.59001 (9)	0.0547 (4)	
S1	0.61709 (3)	0.10285 (10)	0.56935 (3)	0.04091 (17)	

### (E)-N'-[4-(Piperidin-1-yl)benzylidene]benzenesulfonohydrazide (I) Atomic displacement parameters (Å^2^)

**Table:**

	*U*^11^	*U*^22^	*U*^33^	*U*^12^	*U*^13^	*U*^23^
C1	0.0281 (10)	0.0418 (12)	0.0442 (11)	−0.0031 (9)	0.0075 (8)	−0.0030 (9)
C2	0.0554 (14)	0.0543 (14)	0.0594 (15)	0.0026 (12)	0.0210 (11)	−0.0046 (12)
C3	0.0813 (19)	0.083 (2)	0.0578 (16)	−0.0118 (17)	0.0297 (14)	−0.0194 (16)
C4	0.0640 (16)	0.102 (2)	0.0386 (13)	−0.0277 (17)	0.0046 (12)	−0.0034 (15)
C5	0.0536 (15)	0.089 (2)	0.0564 (16)	0.0059 (15)	−0.0001 (12)	0.0206 (15)
C6	0.0480 (13)	0.0631 (16)	0.0524 (14)	0.0131 (12)	0.0103 (10)	0.0074 (12)
C7	0.0473 (12)	0.0377 (12)	0.0347 (11)	0.0034 (10)	0.0089 (9)	0.0014 (9)
C8	0.0426 (11)	0.0356 (11)	0.0324 (10)	−0.0006 (9)	0.0050 (8)	0.0045 (9)
C9	0.0443 (12)	0.0349 (11)	0.0459 (12)	−0.0086 (10)	0.0033 (9)	−0.0063 (10)
C10	0.0465 (12)	0.0318 (11)	0.0483 (12)	−0.0028 (10)	0.0079 (10)	−0.0092 (10)
C11	0.0432 (11)	0.0312 (10)	0.0278 (9)	−0.0037 (9)	0.0027 (8)	0.0018 (8)
C12	0.0503 (12)	0.0339 (11)	0.0415 (11)	−0.0107 (10)	0.0073 (9)	−0.0086 (9)
C13	0.0523 (12)	0.0352 (11)	0.0430 (11)	−0.0014 (10)	0.0126 (9)	−0.0063 (10)
C14	0.0505 (13)	0.0579 (15)	0.0492 (13)	−0.0097 (11)	0.0116 (10)	−0.0172 (12)
C15	0.0519 (14)	0.0626 (17)	0.0857 (19)	−0.0081 (13)	0.0235 (13)	−0.0283 (15)
C16	0.0453 (13)	0.0577 (15)	0.0657 (15)	−0.0070 (12)	0.0171 (11)	−0.0130 (12)
C17	0.0481 (13)	0.0739 (18)	0.0535 (14)	−0.0195 (13)	0.0082 (11)	−0.0148 (13)
C18	0.0536 (14)	0.0606 (16)	0.0598 (15)	−0.0205 (12)	0.0191 (11)	−0.0261 (12)
N1	0.0397 (10)	0.0413 (10)	0.0444 (10)	0.0028 (8)	0.0089 (8)	−0.0016 (9)
N2	0.0382 (9)	0.0433 (10)	0.0393 (9)	0.0021 (8)	0.0055 (7)	0.0022 (8)
N3	0.0422 (9)	0.0387 (10)	0.0330 (9)	−0.0091 (8)	0.0062 (7)	−0.0053 (7)
O1	0.0619 (10)	0.0391 (9)	0.0689 (11)	0.0043 (8)	0.0147 (8)	0.0115 (8)
O2	0.0444 (9)	0.0671 (11)	0.0572 (9)	0.0011 (8)	0.0216 (7)	0.0009 (8)
S1	0.0378 (3)	0.0397 (3)	0.0456 (3)	0.0016 (2)	0.0118 (2)	0.0042 (2)

### (E)-N'-[4-(Piperidin-1-yl)benzylidene]benzenesulfonohydrazide (I) Geometric parameters (Å, º)

**Table:**

C1—C2	1.370 (3)	C12—H12	0.9300
C1—C6	1.383 (3)	C13—H13	0.9300
C1—S1	1.758 (2)	C14—N3	1.460 (3)
C2—C3	1.381 (4)	C14—C15	1.507 (3)
C2—H2	0.9300	C14—H14A	0.9700
C3—C4	1.363 (4)	C14—H14B	0.9700
C3—H3	0.9300	C15—C16	1.503 (3)
C4—C5	1.370 (4)	C15—H15A	0.9700
C4—H4	0.9300	C15—H15B	0.9700
C5—C6	1.382 (3)	C16—C17	1.500 (3)
C5—H5	0.9300	C16—H16A	0.9700
C6—H6	0.9300	C16—H16B	0.9700
C7—N2	1.271 (3)	C17—C18	1.509 (3)
C7—C8	1.458 (3)	C17—H17A	0.9700
C7—H7	0.9300	C17—H17B	0.9700
C8—C13	1.387 (3)	C18—N3	1.463 (3)
C8—C9	1.395 (3)	C18—H18A	0.9700
C9—C10	1.372 (3)	C18—H18B	0.9700
C9—H9	0.9300	N1—N2	1.397 (2)
C10—C11	1.406 (3)	N1—S1	1.6458 (19)
C10—H10	0.9300	N1—H1N	0.84 (2)
C11—C12	1.397 (3)	O1—S1	1.4258 (16)
C11—N3	1.402 (2)	O2—S1	1.4216 (15)
C12—C13	1.379 (3)		
			
C2—C1—C6	121.3 (2)	C15—C14—H14A	108.9
C2—C1—S1	120.43 (17)	N3—C14—H14B	108.9
C6—C1—S1	118.24 (17)	C15—C14—H14B	108.9
C1—C2—C3	118.8 (2)	H14A—C14—H14B	107.7
C1—C2—H2	120.6	C16—C15—C14	112.1 (2)
C3—C2—H2	120.6	C16—C15—H15A	109.2
C4—C3—C2	120.8 (3)	C14—C15—H15A	109.2
C4—C3—H3	119.6	C16—C15—H15B	109.2
C2—C3—H3	119.6	C14—C15—H15B	109.2
C3—C4—C5	120.1 (2)	H15A—C15—H15B	107.9
C3—C4—H4	119.9	C17—C16—C15	108.8 (2)
C5—C4—H4	119.9	C17—C16—H16A	109.9
C4—C5—C6	120.4 (3)	C15—C16—H16A	109.9
C4—C5—H5	119.8	C17—C16—H16B	109.9
C6—C5—H5	119.8	C15—C16—H16B	109.9
C5—C6—C1	118.6 (2)	H16A—C16—H16B	108.3
C5—C6—H6	120.7	C16—C17—C18	111.60 (19)
C1—C6—H6	120.7	C16—C17—H17A	109.3
N2—C7—C8	122.39 (19)	C18—C17—H17A	109.3
N2—C7—H7	118.8	C16—C17—H17B	109.3
C8—C7—H7	118.8	C18—C17—H17B	109.3
C13—C8—C9	116.90 (19)	H17A—C17—H17B	108.0
C13—C8—C7	119.79 (19)	N3—C18—C17	112.8 (2)
C9—C8—C7	123.29 (19)	N3—C18—H18A	109.0
C10—C9—C8	121.45 (19)	C17—C18—H18A	109.0
C10—C9—H9	119.3	N3—C18—H18B	109.0
C8—C9—H9	119.3	C17—C18—H18B	109.0
C9—C10—C11	121.9 (2)	H18A—C18—H18B	107.8
C9—C10—H10	119.1	N2—N1—S1	115.68 (14)
C11—C10—H10	119.1	N2—N1—H1N	116.4 (17)
C12—C11—N3	122.65 (18)	S1—N1—H1N	114.2 (16)
C12—C11—C10	116.36 (19)	C7—N2—N1	115.09 (18)
N3—C11—C10	120.96 (18)	C11—N3—C14	116.53 (15)
C13—C12—C11	121.31 (19)	C11—N3—C18	116.14 (17)
C13—C12—H12	119.3	C14—N3—C18	113.21 (17)
C11—C12—H12	119.3	O2—S1—O1	120.46 (10)
C12—C13—C8	122.1 (2)	O2—S1—N1	103.75 (10)
C12—C13—H13	119.0	O1—S1—N1	107.20 (10)
C8—C13—H13	119.0	O2—S1—C1	108.99 (9)
N3—C14—C15	113.40 (18)	O1—S1—C1	108.67 (10)
N3—C14—H14A	108.9	N1—S1—C1	106.95 (9)
			
C6—C1—C2—C3	−0.5 (3)	C15—C16—C17—C18	−56.4 (3)
S1—C1—C2—C3	−179.69 (19)	C16—C17—C18—N3	54.7 (3)
C1—C2—C3—C4	0.6 (4)	C8—C7—N2—N1	176.43 (17)
C2—C3—C4—C5	−0.8 (4)	S1—N1—N2—C7	167.78 (14)
C3—C4—C5—C6	0.8 (4)	C12—C11—N3—C14	−142.9 (2)
C4—C5—C6—C1	−0.7 (4)	C10—C11—N3—C14	39.3 (3)
C2—C1—C6—C5	0.5 (3)	C12—C11—N3—C18	−5.6 (3)
S1—C1—C6—C5	179.75 (18)	C10—C11—N3—C18	176.68 (19)
N2—C7—C8—C13	−172.29 (19)	C15—C14—N3—C11	−172.2 (2)
N2—C7—C8—C9	6.1 (3)	C15—C14—N3—C18	49.3 (3)
C13—C8—C9—C10	−1.2 (3)	C17—C18—N3—C11	170.97 (18)
C7—C8—C9—C10	−179.67 (19)	C17—C18—N3—C14	−50.3 (3)
C8—C9—C10—C11	0.1 (3)	N2—N1—S1—O2	178.88 (14)
C9—C10—C11—C12	1.1 (3)	N2—N1—S1—O1	50.42 (17)
C9—C10—C11—N3	178.96 (18)	N2—N1—S1—C1	−65.99 (16)
N3—C11—C12—C13	−178.92 (18)	C2—C1—S1—O2	−145.87 (18)
C10—C11—C12—C13	−1.1 (3)	C6—C1—S1—O2	34.91 (19)
C11—C12—C13—C8	−0.1 (3)	C2—C1—S1—O1	−12.9 (2)
C9—C8—C13—C12	1.2 (3)	C6—C1—S1—O1	167.93 (16)
C7—C8—C13—C12	179.73 (19)	C2—C1—S1—N1	102.56 (19)
N3—C14—C15—C16	−52.5 (3)	C6—C1—S1—N1	−76.65 (18)
C14—C15—C16—C17	55.3 (3)		

### (E)-N'-[4-(Piperidin-1-yl)benzylidene]benzenesulfonohydrazide (I) Hydrogen-bond geometry (Å, º)

**Table:**

*D*—H···*A*	*D*—H	H···*A*	*D*···*A*	*D*—H···*A*
N1—H1*N*···O1^i^	0.84 (2)	2.32 (2)	3.133 (3)	165 (2)

Symmetry code: (i) *x*, *y*+1, *z*.

### (E)-4-Methyl-N'-[4-(piperidin-1-yl)benzylidene]benzenesulfonohydrazide (II) Crystal data

**Table:**

C_19_H_23_N_3_O_2_S	*F*(000) = 760
*M**_r_* = 357.46	*D*_x_ = 1.272 Mg m^−^^3^
Monoclinic, *P*2_1_/*c*	Mo *K*α radiation, λ = 0.71073 Å
*a* = 18.442 (2) Å	Cell parameters from 1748 reflections
*b* = 5.3250 (4) Å	θ = 2.6–28.0°
*c* = 19.412 (2) Å	µ = 0.19 mm^−^^1^
β = 101.74 (1)°	*T* = 293 K
*V* = 1866.5 (3) Å^3^	Prism, light pink
*Z* = 4	0.48 × 0.24 × 0.10 mm

### (E)-4-Methyl-N'-[4-(piperidin-1-yl)benzylidene]benzenesulfonohydrazide (II) Data collection

**Table:**

Oxford Diffraction Xcalibur diffractometer with Sapphire CCD	2166 reflections with *I* > 2σ(*I*)
Radiation source: Enhance (Mo) X-ray Source	*R*_int_ = 0.026
Rotation method data acquisition using ω scans.	θ_max_ = 25.4°, θ_min_ = 2.6°
Absorption correction: multi-scan (CrysAlis RED; Oxford Diffraction, 2009)	*h* = −22→13
*T*_min_ = 0.914, *T*_max_ = 0.981	*k* = −6→2
6735 measured reflections	*l* = −21→23
3422 independent reflections	

### (E)-4-Methyl-N'-[4-(piperidin-1-yl)benzylidene]benzenesulfonohydrazide (II) Refinement

**Table:**

Refinement on *F*^2^	0 restraints
Least-squares matrix: full	Hydrogen site location: mixed
*R*[*F*^2^ > 2σ(*F*^2^)] = 0.051	H atoms treated by a mixture of independent and constrained refinement
*wR*(*F*^2^) = 0.137	*w* = 1/[σ^2^(*F*_o_^2^) + (0.0561*P*)^2^ + 0.7083*P*] where *P* = (*F*_o_^2^ + 2*F*_c_^2^)/3
*S* = 1.02	(Δ/σ)_max_ < 0.001
3422 reflections	Δρ_max_ = 0.21 e Å^−^^3^
230 parameters	Δρ_min_ = −0.22 e Å^−^^3^

### (E)-4-Methyl-N'-[4-(piperidin-1-yl)benzylidene]benzenesulfonohydrazide (II) Special details

**Table:**

Geometry. All esds (except the esd in the dihedral angle between two l.s. planes) are estimated using the full covariance matrix. The cell esds are taken into account individually in the estimation of esds in distances, angles and torsion angles; correlations between esds in cell parameters are only used when they are defined by crystal symmetry. An approximate (isotropic) treatment of cell esds is used for estimating esds involving l.s. planes.

### (E)-4-Methyl-N'-[4-(piperidin-1-yl)benzylidene]benzenesulfonohydrazide (II) Fractional atomic coordinates and isotropic or equivalent isotropic displacement parameters (Å^2^)

**Table:**

	*x*	*y*	*z*	*U*_iso_*/*U*_eq_	
C1	0.87009 (13)	0.2262 (5)	0.74497 (13)	0.0464 (6)	
C2	0.85130 (16)	0.0568 (6)	0.69097 (15)	0.0610 (8)	
H2	0.8189	−0.0742	0.6944	0.073*	
C3	0.88094 (18)	0.0827 (6)	0.63160 (16)	0.0716 (9)	
H3	0.8682	−0.0331	0.5953	0.086*	
C4	0.92855 (15)	0.2738 (6)	0.62451 (15)	0.0640 (8)	
C5	0.94574 (15)	0.4437 (6)	0.67864 (16)	0.0635 (8)	
H5	0.9775	0.5760	0.6746	0.076*	
C6	0.91734 (15)	0.4236 (5)	0.73839 (15)	0.0579 (7)	
H6	0.9296	0.5414	0.7742	0.070*	
C7	0.65696 (15)	0.5049 (5)	0.75489 (14)	0.0549 (7)	
H7	0.6648	0.6537	0.7804	0.066*	
C8	0.58859 (14)	0.4736 (5)	0.70327 (14)	0.0497 (7)	
C9	0.57536 (16)	0.2675 (5)	0.65812 (16)	0.0606 (8)	
H9	0.6120	0.1463	0.6600	0.073*	
C10	0.50985 (16)	0.2394 (5)	0.61123 (16)	0.0603 (8)	
H10	0.5032	0.0999	0.5818	0.072*	
C11	0.45234 (15)	0.4149 (5)	0.60617 (14)	0.0508 (7)	
C12	0.46593 (15)	0.6186 (5)	0.65168 (15)	0.0570 (7)	
H12	0.4293	0.7396	0.6503	0.068*	
C13	0.53232 (15)	0.6458 (5)	0.69881 (15)	0.0567 (7)	
H13	0.5392	0.7848	0.7284	0.068*	
C14	0.38983 (18)	0.2897 (8)	0.48900 (18)	0.0869 (11)	
H14A	0.4194	0.1377	0.4942	0.104*	
H14B	0.4156	0.4144	0.4665	0.104*	
C15	0.3168 (2)	0.2351 (7)	0.44174 (19)	0.0947 (12)	
H15A	0.3246	0.2074	0.3944	0.114*	
H15B	0.2970	0.0811	0.4573	0.114*	
C16	0.2620 (2)	0.4358 (7)	0.43997 (19)	0.0892 (11)	
H16A	0.2143	0.3791	0.4139	0.107*	
H16B	0.2767	0.5809	0.4159	0.107*	
C17	0.25546 (19)	0.5082 (9)	0.5121 (2)	0.1010 (13)	
H17A	0.2317	0.3727	0.5326	0.121*	
H17B	0.2239	0.6550	0.5095	0.121*	
C18	0.32853 (18)	0.5650 (8)	0.55932 (19)	0.0931 (12)	
H18A	0.3460	0.7256	0.5456	0.112*	
H18B	0.3210	0.5817	0.6071	0.112*	
C19	0.9621 (2)	0.2953 (9)	0.56057 (18)	0.1048 (13)	
H19A	1.0151	0.2897	0.5744	0.157*	
H19B	0.9474	0.4515	0.5372	0.157*	
H19C	0.9453	0.1585	0.5292	0.157*	
N1	0.76763 (13)	0.3858 (4)	0.81973 (13)	0.0564 (6)	
H1N	0.7786 (17)	0.529 (6)	0.8265 (15)	0.068*	
N2	0.70639 (12)	0.3358 (4)	0.76625 (12)	0.0541 (6)	
N3	0.38501 (12)	0.3803 (4)	0.55849 (12)	0.0592 (6)	
O1	0.89223 (11)	0.2837 (4)	0.87969 (10)	0.0729 (6)	
O2	0.80891 (11)	−0.0554 (4)	0.82456 (11)	0.0711 (6)	
S1	0.83734 (4)	0.19279 (14)	0.82295 (4)	0.0548 (2)	

### (E)-4-Methyl-N'-[4-(piperidin-1-yl)benzylidene]benzenesulfonohydrazide (II) Atomic displacement parameters (Å^2^)

**Table:**

	*U*^11^	*U*^22^	*U*^33^	*U*^12^	*U*^13^	*U*^23^
C1	0.0376 (13)	0.0478 (15)	0.0508 (15)	0.0021 (12)	0.0023 (11)	0.0067 (13)
C2	0.0635 (18)	0.0558 (18)	0.0625 (19)	−0.0139 (15)	0.0102 (15)	−0.0011 (16)
C3	0.078 (2)	0.075 (2)	0.0595 (19)	−0.0078 (19)	0.0097 (16)	−0.0107 (17)
C4	0.0498 (17)	0.081 (2)	0.0595 (19)	0.0021 (17)	0.0074 (14)	0.0110 (17)
C5	0.0476 (16)	0.073 (2)	0.069 (2)	−0.0149 (15)	0.0094 (15)	0.0132 (17)
C6	0.0514 (16)	0.0578 (17)	0.0609 (18)	−0.0082 (14)	0.0027 (14)	−0.0007 (15)
C7	0.0570 (17)	0.0475 (16)	0.0638 (18)	−0.0020 (15)	0.0205 (14)	0.0029 (14)
C8	0.0489 (16)	0.0415 (15)	0.0630 (17)	0.0007 (13)	0.0212 (13)	0.0071 (13)
C9	0.0555 (18)	0.0483 (18)	0.081 (2)	0.0126 (14)	0.0214 (15)	−0.0012 (15)
C10	0.0601 (18)	0.0470 (17)	0.075 (2)	0.0066 (14)	0.0166 (15)	−0.0100 (14)
C11	0.0536 (16)	0.0468 (16)	0.0575 (17)	0.0041 (13)	0.0244 (14)	0.0025 (14)
C12	0.0540 (17)	0.0523 (17)	0.0678 (18)	0.0154 (14)	0.0196 (15)	−0.0018 (14)
C13	0.0601 (18)	0.0447 (16)	0.0673 (19)	0.0057 (14)	0.0178 (15)	−0.0046 (14)
C14	0.075 (2)	0.106 (3)	0.080 (2)	0.018 (2)	0.0142 (18)	−0.019 (2)
C15	0.094 (3)	0.100 (3)	0.082 (3)	0.014 (2)	−0.003 (2)	−0.026 (2)
C16	0.080 (2)	0.087 (3)	0.092 (3)	0.008 (2)	−0.004 (2)	−0.012 (2)
C17	0.062 (2)	0.146 (4)	0.092 (3)	0.024 (2)	0.0066 (19)	−0.027 (3)
C18	0.062 (2)	0.120 (3)	0.091 (3)	0.027 (2)	0.0031 (18)	−0.037 (2)
C19	0.096 (3)	0.157 (4)	0.068 (2)	−0.011 (3)	0.034 (2)	0.010 (2)
N1	0.0554 (15)	0.0502 (14)	0.0646 (15)	−0.0005 (12)	0.0141 (12)	0.0008 (13)
N2	0.0468 (13)	0.0513 (14)	0.0650 (15)	−0.0022 (12)	0.0133 (11)	0.0058 (12)
N3	0.0522 (14)	0.0670 (16)	0.0603 (15)	0.0086 (12)	0.0161 (11)	−0.0056 (12)
O1	0.0673 (13)	0.0926 (16)	0.0530 (12)	0.0007 (12)	−0.0013 (10)	0.0053 (11)
O2	0.0852 (15)	0.0493 (12)	0.0840 (15)	−0.0002 (11)	0.0293 (12)	0.0194 (10)
S1	0.0529 (4)	0.0546 (4)	0.0558 (4)	0.0012 (4)	0.0083 (3)	0.0101 (4)

### (E)-4-Methyl-N'-[4-(piperidin-1-yl)benzylidene]benzenesulfonohydrazide (II) Geometric parameters (Å, º)

**Table:**

C1—C2	1.373 (4)	C13—H13	0.9300
C1—C6	1.388 (4)	C14—N3	1.452 (4)
C1—S1	1.749 (3)	C14—C15	1.496 (4)
C2—C3	1.379 (4)	C14—H14A	0.9700
C2—H2	0.9300	C14—H14B	0.9700
C3—C4	1.369 (4)	C15—C16	1.466 (5)
C3—H3	0.9300	C15—H15A	0.9700
C4—C5	1.374 (4)	C15—H15B	0.9700
C4—C19	1.499 (4)	C16—C17	1.481 (5)
C5—C6	1.370 (4)	C16—H16A	0.9700
C5—H5	0.9300	C16—H16B	0.9700
C6—H6	0.9300	C17—C18	1.498 (4)
C7—N2	1.269 (3)	C17—H17A	0.9700
C7—C8	1.452 (4)	C17—H17B	0.9700
C7—H7	0.9300	C18—N3	1.435 (4)
C8—C13	1.374 (4)	C18—H18A	0.9700
C8—C9	1.395 (4)	C18—H18B	0.9700
C9—C10	1.365 (4)	C19—H19A	0.9600
C9—H9	0.9300	C19—H19B	0.9600
C10—C11	1.402 (4)	C19—H19C	0.9600
C10—H10	0.9300	N1—N2	1.395 (3)
C11—C12	1.389 (4)	N1—S1	1.637 (3)
C11—N3	1.402 (3)	N1—H1N	0.79 (3)
C12—C13	1.379 (4)	O1—S1	1.421 (2)
C12—H12	0.9300	O2—S1	1.424 (2)
			
C2—C1—C6	119.5 (3)	H14A—C14—H14B	107.6
C2—C1—S1	121.1 (2)	C16—C15—C14	113.7 (3)
C6—C1—S1	119.4 (2)	C16—C15—H15A	108.8
C1—C2—C3	119.4 (3)	C14—C15—H15A	108.8
C1—C2—H2	120.3	C16—C15—H15B	108.8
C3—C2—H2	120.3	C14—C15—H15B	108.8
C4—C3—C2	122.0 (3)	H15A—C15—H15B	107.7
C4—C3—H3	119.0	C15—C16—C17	110.9 (3)
C2—C3—H3	119.0	C15—C16—H16A	109.5
C3—C4—C5	117.7 (3)	C17—C16—H16A	109.5
C3—C4—C19	121.4 (3)	C15—C16—H16B	109.5
C5—C4—C19	120.9 (3)	C17—C16—H16B	109.5
C6—C5—C4	121.9 (3)	H16A—C16—H16B	108.1
C6—C5—H5	119.1	C16—C17—C18	113.2 (3)
C4—C5—H5	119.1	C16—C17—H17A	108.9
C5—C6—C1	119.5 (3)	C18—C17—H17A	108.9
C5—C6—H6	120.3	C16—C17—H17B	108.9
C1—C6—H6	120.3	C18—C17—H17B	108.9
N2—C7—C8	122.1 (3)	H17A—C17—H17B	107.7
N2—C7—H7	119.0	N3—C18—C17	114.8 (3)
C8—C7—H7	119.0	N3—C18—H18A	108.6
C13—C8—C9	116.9 (3)	C17—C18—H18A	108.6
C13—C8—C7	120.4 (3)	N3—C18—H18B	108.6
C9—C8—C7	122.7 (2)	C17—C18—H18B	108.6
C10—C9—C8	121.5 (3)	H18A—C18—H18B	107.5
C10—C9—H9	119.2	C4—C19—H19A	109.5
C8—C9—H9	119.2	C4—C19—H19B	109.5
C9—C10—C11	121.9 (3)	H19A—C19—H19B	109.5
C9—C10—H10	119.1	C4—C19—H19C	109.5
C11—C10—H10	119.1	H19A—C19—H19C	109.5
C12—C11—N3	122.9 (2)	H19B—C19—H19C	109.5
C12—C11—C10	116.1 (3)	N2—N1—S1	114.78 (19)
N3—C11—C10	121.0 (3)	N2—N1—H1N	117 (2)
C13—C12—C11	121.7 (2)	S1—N1—H1N	115 (2)
C13—C12—H12	119.2	C7—N2—N1	116.0 (2)
C11—C12—H12	119.2	C11—N3—C18	116.7 (2)
C8—C13—C12	121.9 (3)	C11—N3—C14	116.3 (2)
C8—C13—H13	119.0	C18—N3—C14	114.8 (2)
C12—C13—H13	119.0	O1—S1—O2	120.42 (13)
N3—C14—C15	114.6 (3)	O1—S1—N1	104.23 (13)
N3—C14—H14A	108.6	O2—S1—N1	107.07 (13)
C15—C14—H14A	108.6	O1—S1—C1	108.61 (12)
N3—C14—H14B	108.6	O2—S1—C1	107.88 (13)
C15—C14—H14B	108.6	N1—S1—C1	108.05 (12)
			
C6—C1—C2—C3	1.3 (4)	C14—C15—C16—C17	−51.1 (5)
S1—C1—C2—C3	−177.0 (2)	C15—C16—C17—C18	51.3 (5)
C1—C2—C3—C4	−0.3 (5)	C16—C17—C18—N3	−48.4 (5)
C2—C3—C4—C5	−0.7 (4)	C8—C7—N2—N1	−176.9 (2)
C2—C3—C4—C19	178.2 (3)	S1—N1—N2—C7	−168.48 (19)
C3—C4—C5—C6	0.7 (4)	C12—C11—N3—C18	−1.2 (4)
C19—C4—C5—C6	−178.2 (3)	C10—C11—N3—C18	177.5 (3)
C4—C5—C6—C1	0.3 (4)	C12—C11—N3—C14	139.5 (3)
C2—C1—C6—C5	−1.3 (4)	C10—C11—N3—C14	−41.8 (4)
S1—C1—C6—C5	177.1 (2)	C17—C18—N3—C11	−174.8 (3)
N2—C7—C8—C13	171.5 (3)	C17—C18—N3—C14	43.9 (4)
N2—C7—C8—C9	−6.2 (4)	C15—C14—N3—C11	175.2 (3)
C13—C8—C9—C10	0.6 (4)	C15—C14—N3—C18	−43.4 (4)
C7—C8—C9—C10	178.4 (3)	N2—N1—S1—O1	178.95 (18)
C8—C9—C10—C11	−0.4 (4)	N2—N1—S1—O2	−52.4 (2)
C9—C10—C11—C12	−0.1 (4)	N2—N1—S1—C1	63.5 (2)
C9—C10—C11—N3	−178.9 (3)	C2—C1—S1—O1	146.9 (2)
N3—C11—C12—C13	179.0 (3)	C6—C1—S1—O1	−31.4 (2)
C10—C11—C12—C13	0.2 (4)	C2—C1—S1—O2	14.9 (2)
C9—C8—C13—C12	−0.5 (4)	C6—C1—S1—O2	−163.5 (2)
C7—C8—C13—C12	−178.3 (3)	C2—C1—S1—N1	−100.6 (2)
C11—C12—C13—C8	0.1 (4)	C6—C1—S1—N1	81.1 (2)
N3—C14—C15—C16	47.7 (5)		

### (E)-4-Methyl-N'-[4-(piperidin-1-yl)benzylidene]benzenesulfonohydrazide (II) Hydrogen-bond geometry (Å, º)

**Table:**

*D*—H···*A*	*D*—H	H···*A*	*D*···*A*	*D*—H···*A*
N1—H1*N*···O2^i^	0.79 (3)	2.29 (3)	3.068 (3)	170 (3)

Symmetry code: (i) *x*, *y*+1, *z*.

### (E)-4-Chloro-N'-[4-(piperidin-1-yl)benzylidene]benzenesulfonohydrazide (III) Crystal data

**Table:**

C_18_H_20_ClN_3_O_2_S	*F*(000) = 3168
*M**_r_* = 377.88	*D*_x_ = 1.346 Mg m^−^^3^
Monoclinic, *C*2/*c*	Mo *K*α radiation, λ = 0.71073 Å
*a* = 33.052 (6) Å	Cell parameters from 1174 reflections
*b* = 5.258 (1) Å	θ = 2.5–27.8°
*c* = 43.026 (8) Å	µ = 0.33 mm^−^^1^
β = 94.05 (2)°	*T* = 293 K
*V* = 7459 (2) Å^3^	Prism, red
*Z* = 16	0.50 × 0.26 × 0.14 mm

### (E)-4-Chloro-N'-[4-(piperidin-1-yl)benzylidene]benzenesulfonohydrazide (III) Data collection

**Table:**

Oxford Diffraction Xcalibur diffractometer with Sapphire CCD	2999 reflections with *I* > 2σ(*I*)
Radiation source: Enhance (Mo) X-ray Source	*R*_int_ = 0.042
Rotation method data acquisition using ω scans.	θ_max_ = 25.4°, θ_min_ = 2.7°
Absorption correction: multi-scan (CrysAlis RED; Oxford Diffraction, 2009)	*h* = −39→39
*T*_min_ = 0.851, *T*_max_ = 0.955	*k* = −6→6
14002 measured reflections	*l* = −42→51
6833 independent reflections	

### (E)-4-Chloro-N'-[4-(piperidin-1-yl)benzylidene]benzenesulfonohydrazide (III) Refinement

**Table:**

Refinement on *F*^2^	31 restraints
Least-squares matrix: full	Hydrogen site location: mixed
*R*[*F*^2^ > 2σ(*F*^2^)] = 0.075	H atoms treated by a mixture of independent and constrained refinement
*wR*(*F*^2^) = 0.172	*w* = 1/[σ^2^(*F*_o_^2^) + (0.0483*P*)^2^ + 15.4136*P*] where *P* = (*F*_o_^2^ + 2*F*_c_^2^)/3
*S* = 1.01	(Δ/σ)_max_ < 0.001
6833 reflections	Δρ_max_ = 0.27 e Å^−^^3^
457 parameters	Δρ_min_ = −0.30 e Å^−^^3^

### (E)-4-Chloro-N'-[4-(piperidin-1-yl)benzylidene]benzenesulfonohydrazide (III) Special details

**Table:**

Geometry. All esds (except the esd in the dihedral angle between two l.s. planes) are estimated using the full covariance matrix. The cell esds are taken into account individually in the estimation of esds in distances, angles and torsion angles; correlations between esds in cell parameters are only used when they are defined by crystal symmetry. An approximate (isotropic) treatment of cell esds is used for estimating esds involving l.s. planes.

### (E)-4-Chloro-N'-[4-(piperidin-1-yl)benzylidene]benzenesulfonohydrazide (III) Fractional atomic coordinates and isotropic or equivalent isotropic displacement parameters (Å^2^)

**Table:**

	*x*	*y*	*z*	*U*_iso_*/*U*_eq_	
Cl1	0.47550 (5)	0.3259 (4)	0.18187 (4)	0.1273 (7)	
S1	0.31044 (4)	0.0442 (3)	0.11230 (3)	0.0657 (4)	
O1	0.27796 (10)	0.1162 (6)	0.13046 (7)	0.0779 (10)	
O2	0.31308 (11)	−0.2078 (6)	0.10041 (8)	0.0797 (10)	
N1	0.30660 (14)	0.2366 (8)	0.08245 (9)	0.0636 (12)	
H1N	0.2999 (15)	0.387 (9)	0.0854 (11)	0.076*	
N2	0.33782 (12)	0.2115 (8)	0.06209 (9)	0.0625 (11)	
N3	0.45666 (13)	0.3870 (8)	−0.04820 (10)	0.0770 (12)	
C1	0.35682 (15)	0.1126 (9)	0.13273 (10)	0.0563 (13)	
C2	0.39197 (19)	−0.0048 (11)	0.12540 (13)	0.0843 (17)	
H2	0.3912	−0.1276	0.1098	0.101*	
C3	0.42848 (19)	0.0587 (12)	0.14107 (15)	0.0940 (19)	
H3	0.4523	−0.0232	0.1365	0.113*	
C4	0.42913 (17)	0.2419 (12)	0.16323 (12)	0.0763 (16)	
C5	0.3948 (2)	0.3541 (12)	0.17136 (13)	0.0895 (18)	
H5	0.3958	0.4727	0.1874	0.107*	
C6	0.35822 (17)	0.2929 (11)	0.15586 (12)	0.0813 (16)	
H6	0.3346	0.3735	0.1610	0.098*	
C7	0.34159 (15)	0.3963 (10)	0.04343 (11)	0.0619 (13)	
H7	0.3245	0.5362	0.0445	0.074*	
C8	0.37184 (15)	0.3948 (9)	0.02046 (10)	0.0565 (13)	
C9	0.40063 (17)	0.2080 (10)	0.01923 (12)	0.0733 (15)	
H9	0.4013	0.0785	0.0340	0.088*	
C10	0.42840 (16)	0.2054 (10)	−0.00296 (12)	0.0726 (15)	
H10	0.4473	0.0747	−0.0031	0.087*	
C11	0.42853 (15)	0.3964 (10)	−0.02534 (11)	0.0589 (13)	
C12	0.39956 (16)	0.5837 (10)	−0.02398 (11)	0.0686 (14)	
H12	0.3986	0.7135	−0.0387	0.082*	
C13	0.37211 (16)	0.5843 (9)	−0.00157 (11)	0.0689 (14)	
H13	0.3533	0.7155	−0.0012	0.083*	
C14	0.49585 (19)	0.3073 (16)	−0.04105 (16)	0.143 (3)	
H14A	0.4943	0.1452	−0.0304	0.172*	
H14B	0.5081	0.4269	−0.0260	0.172*	
C15	0.5239 (2)	0.2760 (15)	−0.06531 (18)	0.139 (3)	
H15A	0.5512	0.3004	−0.0560	0.166*	
H15B	0.5220	0.1017	−0.0727	0.166*	
C16	0.5182 (2)	0.4427 (14)	−0.09203 (15)	0.118 (2)	
H16A	0.5346	0.5939	−0.0881	0.142*	
H16B	0.5282	0.3573	−0.1100	0.142*	
C17	0.4780 (2)	0.5174 (17)	−0.09945 (16)	0.150 (3)	
H17A	0.4658	0.3929	−0.1139	0.180*	
H17B	0.4787	0.6774	−0.1106	0.180*	
C18	0.45054 (18)	0.5500 (13)	−0.07439 (13)	0.115 (2)	
H18A	0.4528	0.7243	−0.0671	0.138*	
H18B	0.4230	0.5263	−0.0832	0.138*	
Cl2	0.22664 (5)	0.4803 (4)	0.02015 (4)	0.1245 (7)	
S2	0.13541 (5)	0.7140 (3)	0.13800 (3)	0.0696 (4)	
O3	0.09541 (11)	0.6126 (7)	0.13364 (8)	0.0883 (11)	
O4	0.14190 (11)	0.9734 (6)	0.14659 (7)	0.0845 (11)	
N4	0.15747 (15)	0.5383 (8)	0.16570 (10)	0.0702 (13)	
H4N	0.1518 (16)	0.384 (9)	0.1629 (12)	0.084*	
N5	0.19715 (14)	0.6102 (8)	0.17526 (9)	0.0654 (11)	
N6	0.37521 (14)	0.6426 (8)	0.24681 (10)	0.0726 (12)	
C19	0.16088 (15)	0.6564 (9)	0.10444 (10)	0.0576 (13)	
C20	0.19395 (16)	0.7992 (11)	0.09801 (12)	0.0746 (15)	
H20	0.2028	0.9306	0.1113	0.090*	
C21	0.21407 (16)	0.7463 (12)	0.07161 (13)	0.0823 (17)	
H21	0.2361	0.8446	0.0666	0.099*	
C22	0.20116 (18)	0.5481 (12)	0.05306 (11)	0.0748 (16)	
C23	0.16849 (19)	0.4049 (11)	0.05942 (13)	0.0823 (17)	
H23	0.1600	0.2722	0.0462	0.099*	
C24	0.14812 (17)	0.4565 (10)	0.08532 (12)	0.0771 (16)	
H24	0.1259	0.3583	0.0900	0.093*	
C25	0.21791 (15)	0.4430 (10)	0.19052 (10)	0.0606 (13)	
H25	0.2068	0.2826	0.1932	0.073*	
C26	0.25843 (15)	0.4968 (9)	0.20382 (10)	0.0551 (12)	
C27	0.28111 (17)	0.7029 (10)	0.19542 (10)	0.0654 (14)	
H27	0.2704	0.8106	0.1798	0.078*	
C28	0.31893 (17)	0.7547 (10)	0.20933 (11)	0.0692 (14)	
H28	0.3333	0.8946	0.2028	0.083*	
C29	0.33619 (16)	0.5996 (10)	0.23328 (11)	0.0609 (13)	
C30	0.31319 (16)	0.3937 (10)	0.24176 (11)	0.0675 (14)	
H30	0.3235	0.2879	0.2577	0.081*	
C31	0.27607 (16)	0.3414 (10)	0.22745 (11)	0.0665 (14)	
H31	0.2621	0.1985	0.2335	0.080*	
C32	0.39719 (19)	0.8645 (12)	0.23767 (15)	0.104 (2)	
H32A	0.3844	1.0147	0.2457	0.125*	
H32B	0.3951	0.8759	0.2151	0.125*	
C33	0.4411 (2)	0.8653 (14)	0.24890 (18)	0.123 (2)	
H33A	0.4553	0.7399	0.2372	0.148*	
H33B	0.4525	1.0307	0.2447	0.148*	
C34	0.44807 (19)	0.8091 (14)	0.28267 (17)	0.110 (2)	
H34A	0.4769	0.7871	0.2879	0.132*	
H34B	0.4388	0.9514	0.2946	0.132*	
C35	0.42629 (19)	0.5767 (14)	0.29094 (15)	0.117 (2)	
H35A	0.4279	0.5591	0.3134	0.140*	
H35B	0.4395	0.4302	0.2824	0.140*	
C36	0.38288 (17)	0.5786 (13)	0.27912 (12)	0.099 (2)	
H36A	0.3716	0.4115	0.2825	0.119*	
H36B	0.3685	0.6990	0.2914	0.119*	

### (E)-4-Chloro-N'-[4-(piperidin-1-yl)benzylidene]benzenesulfonohydrazide (III) Atomic displacement parameters (Å^2^)

**Table:**

	*U*^11^	*U*^22^	*U*^33^	*U*^12^	*U*^13^	*U*^23^
Cl1	0.0949 (12)	0.1716 (18)	0.1116 (13)	−0.0191 (13)	−0.0185 (10)	−0.0055 (13)
S1	0.0844 (10)	0.0535 (9)	0.0593 (8)	−0.0097 (8)	0.0064 (7)	0.0000 (7)
O1	0.077 (2)	0.083 (3)	0.076 (2)	−0.012 (2)	0.022 (2)	0.000 (2)
O2	0.108 (3)	0.045 (2)	0.086 (2)	−0.013 (2)	0.002 (2)	−0.0052 (18)
N1	0.083 (3)	0.054 (3)	0.054 (2)	0.005 (3)	0.003 (2)	0.007 (2)
N2	0.080 (3)	0.058 (3)	0.050 (2)	−0.004 (2)	0.004 (2)	−0.002 (2)
N3	0.077 (2)	0.077 (3)	0.077 (3)	0.010 (2)	0.003 (2)	0.017 (2)
C1	0.076 (4)	0.048 (3)	0.046 (3)	−0.007 (3)	0.014 (2)	−0.001 (2)
C2	0.093 (4)	0.080 (4)	0.081 (4)	0.011 (4)	0.010 (4)	−0.025 (3)
C3	0.080 (4)	0.105 (5)	0.098 (5)	0.019 (4)	0.010 (4)	−0.016 (4)
C4	0.077 (4)	0.089 (5)	0.062 (3)	−0.009 (4)	0.004 (3)	0.000 (3)
C5	0.092 (5)	0.101 (5)	0.075 (4)	−0.002 (4)	0.001 (4)	−0.031 (3)
C6	0.083 (4)	0.090 (4)	0.071 (4)	0.006 (4)	0.009 (3)	−0.023 (3)
C7	0.073 (4)	0.058 (3)	0.053 (3)	−0.001 (3)	−0.004 (3)	0.001 (3)
C8	0.070 (3)	0.050 (3)	0.048 (3)	−0.005 (3)	−0.006 (3)	0.001 (3)
C9	0.093 (4)	0.062 (4)	0.064 (3)	0.011 (4)	0.001 (3)	0.021 (3)
C10	0.081 (4)	0.063 (4)	0.074 (4)	0.021 (3)	0.008 (3)	0.015 (3)
C11	0.062 (3)	0.059 (3)	0.056 (3)	0.000 (3)	0.000 (3)	0.002 (3)
C12	0.079 (4)	0.065 (4)	0.062 (3)	0.014 (3)	0.005 (3)	0.019 (3)
C13	0.080 (4)	0.059 (3)	0.068 (3)	0.017 (3)	0.011 (3)	0.013 (3)
C14	0.100 (3)	0.204 (7)	0.128 (5)	0.073 (5)	0.031 (4)	0.061 (5)
C15	0.114 (5)	0.156 (6)	0.152 (6)	0.052 (5)	0.049 (5)	0.043 (5)
C16	0.119 (4)	0.127 (6)	0.113 (5)	0.031 (5)	0.047 (4)	0.026 (5)
C17	0.122 (4)	0.220 (7)	0.115 (4)	0.048 (5)	0.054 (3)	0.062 (5)
C18	0.108 (4)	0.149 (5)	0.091 (4)	0.041 (4)	0.032 (3)	0.054 (3)
Cl2	0.1149 (13)	0.1825 (19)	0.0782 (10)	0.0329 (13)	0.0209 (9)	−0.0211 (12)
S2	0.0840 (11)	0.0686 (10)	0.0560 (8)	0.0118 (9)	0.0033 (7)	0.0047 (7)
O3	0.069 (2)	0.107 (3)	0.088 (3)	0.003 (2)	0.003 (2)	0.009 (2)
O4	0.122 (3)	0.062 (2)	0.070 (2)	0.019 (2)	0.012 (2)	−0.0044 (19)
N4	0.083 (3)	0.068 (3)	0.058 (3)	−0.002 (3)	−0.001 (2)	0.008 (2)
N5	0.080 (3)	0.064 (3)	0.051 (2)	0.005 (3)	−0.002 (2)	0.002 (2)
N6	0.078 (3)	0.074 (3)	0.067 (3)	−0.002 (3)	0.015 (2)	0.013 (2)
C19	0.068 (3)	0.054 (3)	0.049 (3)	0.001 (3)	−0.006 (2)	0.001 (3)
C20	0.081 (4)	0.080 (4)	0.062 (4)	−0.008 (4)	−0.004 (3)	−0.014 (3)
C21	0.076 (4)	0.097 (5)	0.074 (4)	−0.011 (4)	0.004 (3)	0.006 (4)
C22	0.080 (4)	0.090 (4)	0.053 (3)	0.020 (4)	−0.002 (3)	−0.006 (3)
C23	0.105 (5)	0.070 (4)	0.070 (4)	0.000 (4)	−0.005 (4)	−0.012 (3)
C24	0.104 (4)	0.065 (4)	0.063 (3)	−0.019 (3)	0.005 (3)	0.001 (3)
C25	0.072 (4)	0.056 (3)	0.056 (3)	0.002 (3)	0.016 (3)	0.000 (3)
C26	0.072 (4)	0.047 (3)	0.048 (3)	0.005 (3)	0.017 (3)	0.000 (2)
C27	0.085 (4)	0.064 (4)	0.047 (3)	0.006 (3)	0.007 (3)	0.005 (3)
C28	0.090 (4)	0.058 (3)	0.062 (3)	−0.007 (3)	0.021 (3)	0.013 (3)
C29	0.070 (4)	0.064 (4)	0.049 (3)	0.008 (3)	0.015 (3)	−0.005 (3)
C30	0.071 (4)	0.069 (4)	0.064 (3)	0.007 (3)	0.009 (3)	0.021 (3)
C31	0.069 (4)	0.060 (3)	0.073 (3)	0.006 (3)	0.017 (3)	0.015 (3)
C32	0.097 (5)	0.100 (5)	0.114 (5)	−0.016 (4)	−0.001 (4)	0.014 (4)
C33	0.090 (5)	0.136 (6)	0.143 (7)	−0.024 (5)	0.007 (5)	0.022 (5)
C34	0.092 (5)	0.110 (6)	0.127 (6)	−0.009 (5)	−0.009 (4)	−0.012 (5)
C35	0.103 (5)	0.138 (6)	0.107 (5)	−0.017 (5)	−0.015 (4)	0.029 (5)
C36	0.086 (4)	0.143 (6)	0.068 (4)	−0.025 (4)	0.000 (3)	0.006 (4)

### (E)-4-Chloro-N'-[4-(piperidin-1-yl)benzylidene]benzenesulfonohydrazide (III) Geometric parameters (Å, º)

**Table:**

Cl1—C4	1.736 (5)	Cl2—C22	1.735 (5)
S1—O1	1.423 (3)	S2—O4	1.425 (3)
S1—O2	1.425 (3)	S2—O3	1.426 (4)
S1—N1	1.633 (4)	S2—N4	1.638 (4)
S1—C1	1.750 (5)	S2—C19	1.748 (5)
N1—N2	1.406 (5)	N4—N5	1.399 (5)
N1—H1N	0.83 (4)	N4—H4N	0.84 (5)
N2—C7	1.272 (5)	N5—C25	1.269 (5)
N3—C14	1.375 (6)	N6—C29	1.395 (6)
N3—C11	1.401 (6)	N6—C36	1.435 (6)
N3—C18	1.419 (6)	N6—C32	1.444 (6)
C1—C2	1.371 (6)	C19—C20	1.371 (6)
C1—C6	1.373 (6)	C19—C24	1.382 (6)
C2—C3	1.381 (7)	C20—C21	1.384 (7)
C2—H2	0.9300	C20—H20	0.9300
C3—C4	1.355 (7)	C21—C22	1.363 (7)
C3—H3	0.9300	C21—H21	0.9300
C4—C5	1.347 (7)	C22—C23	1.360 (7)
C5—C6	1.377 (7)	C23—C24	1.369 (7)
C5—H5	0.9300	C23—H23	0.9300
C6—H6	0.9300	C24—H24	0.9300
C7—C8	1.455 (6)	C25—C26	1.447 (6)
C7—H7	0.9300	C25—H25	0.9300
C8—C9	1.371 (6)	C26—C27	1.380 (6)
C8—C13	1.376 (6)	C26—C31	1.399 (6)
C9—C10	1.370 (6)	C27—C28	1.374 (6)
C9—H9	0.9300	C27—H27	0.9300
C10—C11	1.392 (6)	C28—C29	1.403 (6)
C10—H10	0.9300	C28—H28	0.9300
C11—C12	1.378 (6)	C29—C30	1.386 (6)
C12—C13	1.370 (6)	C30—C31	1.361 (6)
C12—H12	0.9300	C30—H30	0.9300
C13—H13	0.9300	C31—H31	0.9300
C14—C15	1.453 (7)	C32—C33	1.497 (7)
C14—H14A	0.9700	C32—H32A	0.9700
C14—H14B	0.9700	C32—H32B	0.9700
C15—C16	1.447 (8)	C33—C34	1.484 (8)
C15—H15A	0.9700	C33—H33A	0.9700
C15—H15B	0.9700	C33—H33B	0.9700
C16—C17	1.403 (8)	C34—C35	1.475 (8)
C16—H16A	0.9700	C34—H34A	0.9700
C16—H16B	0.9700	C34—H34B	0.9700
C17—C18	1.467 (7)	C35—C36	1.488 (7)
C17—H17A	0.9700	C35—H35A	0.9700
C17—H17B	0.9700	C35—H35B	0.9700
C18—H18A	0.9700	C36—H36A	0.9700
C18—H18B	0.9700	C36—H36B	0.9700
			
O1—S1—O2	120.8 (2)	O4—S2—O3	120.9 (2)
O1—S1—N1	104.3 (2)	O4—S2—N4	107.5 (2)
O2—S1—N1	107.3 (2)	O3—S2—N4	104.2 (2)
O1—S1—C1	109.7 (2)	O4—S2—C19	107.9 (2)
O2—S1—C1	107.3 (2)	O3—S2—C19	108.8 (2)
N1—S1—C1	106.5 (2)	N4—S2—C19	106.7 (2)
N2—N1—S1	114.3 (3)	N5—N4—S2	114.9 (3)
N2—N1—H1N	114 (4)	N5—N4—H4N	120 (4)
S1—N1—H1N	118 (3)	S2—N4—H4N	111 (4)
C7—N2—N1	115.5 (4)	C25—N5—N4	115.2 (4)
C14—N3—C11	121.0 (5)	C29—N6—C36	117.4 (4)
C14—N3—C18	116.2 (5)	C29—N6—C32	119.0 (5)
C11—N3—C18	118.1 (4)	C36—N6—C32	113.3 (5)
C2—C1—C6	119.4 (5)	C20—C19—C24	120.7 (5)
C2—C1—S1	121.2 (4)	C20—C19—S2	120.6 (4)
C6—C1—S1	119.3 (4)	C24—C19—S2	118.6 (4)
C1—C2—C3	120.3 (5)	C19—C20—C21	119.5 (5)
C1—C2—H2	119.9	C19—C20—H20	120.3
C3—C2—H2	119.9	C21—C20—H20	120.3
C4—C3—C2	119.0 (5)	C22—C21—C20	119.1 (5)
C4—C3—H3	120.5	C22—C21—H21	120.5
C2—C3—H3	120.5	C20—C21—H21	120.5
C5—C4—C3	121.5 (5)	C23—C22—C21	121.7 (5)
C5—C4—Cl1	120.1 (5)	C23—C22—Cl2	119.2 (5)
C3—C4—Cl1	118.4 (5)	C21—C22—Cl2	119.0 (5)
C4—C5—C6	119.9 (5)	C22—C23—C24	119.8 (5)
C4—C5—H5	120.0	C22—C23—H23	120.1
C6—C5—H5	120.0	C24—C23—H23	120.1
C1—C6—C5	119.7 (5)	C23—C24—C19	119.2 (5)
C1—C6—H6	120.1	C23—C24—H24	120.4
C5—C6—H6	120.1	C19—C24—H24	120.4
N2—C7—C8	121.8 (5)	N5—C25—C26	121.4 (5)
N2—C7—H7	119.1	N5—C25—H25	119.3
C8—C7—H7	119.1	C26—C25—H25	119.3
C9—C8—C13	117.0 (5)	C27—C26—C31	116.3 (5)
C9—C8—C7	122.8 (5)	C27—C26—C25	123.7 (5)
C13—C8—C7	120.2 (5)	C31—C26—C25	120.0 (5)
C10—C9—C8	122.3 (5)	C28—C27—C26	122.4 (5)
C10—C9—H9	118.8	C28—C27—H27	118.8
C8—C9—H9	118.8	C26—C27—H27	118.8
C9—C10—C11	120.6 (5)	C27—C28—C29	121.0 (5)
C9—C10—H10	119.7	C27—C28—H28	119.5
C11—C10—H10	119.7	C29—C28—H28	119.5
C12—C11—C10	116.8 (5)	C30—C29—N6	121.7 (5)
C12—C11—N3	123.6 (5)	C30—C29—C28	116.4 (5)
C10—C11—N3	119.6 (5)	N6—C29—C28	121.9 (5)
C13—C12—C11	121.9 (5)	C31—C30—C29	122.2 (5)
C13—C12—H12	119.1	C31—C30—H30	118.9
C11—C12—H12	119.1	C29—C30—H30	118.9
C12—C13—C8	121.3 (5)	C30—C31—C26	121.8 (5)
C12—C13—H13	119.3	C30—C31—H31	119.1
C8—C13—H13	119.3	C26—C31—H31	119.1
N3—C14—C15	120.8 (6)	N6—C32—C33	114.2 (5)
N3—C14—H14A	107.1	N6—C32—H32A	108.7
C15—C14—H14A	107.1	C33—C32—H32A	108.7
N3—C14—H14B	107.1	N6—C32—H32B	108.7
C15—C14—H14B	107.1	C33—C32—H32B	108.7
H14A—C14—H14B	106.8	H32A—C32—H32B	107.6
C16—C15—C14	116.7 (6)	C34—C33—C32	113.3 (6)
C16—C15—H15A	108.1	C34—C33—H33A	108.9
C14—C15—H15A	108.1	C32—C33—H33A	108.9
C16—C15—H15B	108.1	C34—C33—H33B	108.9
C14—C15—H15B	108.1	C32—C33—H33B	108.9
H15A—C15—H15B	107.3	H33A—C33—H33B	107.7
C17—C16—C15	114.7 (6)	C35—C34—C33	110.9 (6)
C17—C16—H16A	108.6	C35—C34—H34A	109.5
C15—C16—H16A	108.6	C33—C34—H34A	109.5
C17—C16—H16B	108.6	C35—C34—H34B	109.5
C15—C16—H16B	108.6	C33—C34—H34B	109.5
H16A—C16—H16B	107.6	H34A—C34—H34B	108.1
C16—C17—C18	119.4 (6)	C34—C35—C36	112.8 (6)
C16—C17—H17A	107.5	C34—C35—H35A	109.0
C18—C17—H17A	107.5	C36—C35—H35A	109.0
C16—C17—H17B	107.5	C34—C35—H35B	109.0
C18—C17—H17B	107.5	C36—C35—H35B	109.0
H17A—C17—H17B	107.0	H35A—C35—H35B	107.8
N3—C18—C17	116.9 (5)	N6—C36—C35	115.6 (5)
N3—C18—H18A	108.1	N6—C36—H36A	108.4
C17—C18—H18A	108.1	C35—C36—H36A	108.4
N3—C18—H18B	108.1	N6—C36—H36B	108.4
C17—C18—H18B	108.1	C35—C36—H36B	108.4
H18A—C18—H18B	107.3	H36A—C36—H36B	107.4
			
O1—S1—N1—N2	175.8 (3)	O4—S2—N4—N5	−47.7 (4)
O2—S1—N1—N2	−55.0 (4)	O3—S2—N4—N5	−177.1 (3)
C1—S1—N1—N2	59.7 (4)	C19—S2—N4—N5	67.8 (4)
S1—N1—N2—C7	−163.0 (3)	S2—N4—N5—C25	−162.9 (3)
O1—S1—C1—C2	159.7 (4)	O4—S2—C19—C20	26.0 (5)
O2—S1—C1—C2	26.7 (5)	O3—S2—C19—C20	158.8 (4)
N1—S1—C1—C2	−87.9 (4)	N4—S2—C19—C20	−89.3 (4)
O1—S1—C1—C6	−22.4 (5)	O4—S2—C19—C24	−156.9 (4)
O2—S1—C1—C6	−155.4 (4)	O3—S2—C19—C24	−24.1 (4)
N1—S1—C1—C6	90.0 (4)	N4—S2—C19—C24	87.8 (4)
C6—C1—C2—C3	−0.3 (8)	C24—C19—C20—C21	1.7 (7)
S1—C1—C2—C3	177.6 (4)	S2—C19—C20—C21	178.7 (4)
C1—C2—C3—C4	−1.4 (9)	C19—C20—C21—C22	−1.9 (8)
C2—C3—C4—C5	3.3 (9)	C20—C21—C22—C23	1.5 (8)
C2—C3—C4—Cl1	−178.1 (5)	C20—C21—C22—Cl2	−179.5 (4)
C3—C4—C5—C6	−3.5 (9)	C21—C22—C23—C24	−1.0 (8)
Cl1—C4—C5—C6	177.9 (4)	Cl2—C22—C23—C24	−180.0 (4)
C2—C1—C6—C5	0.1 (8)	C22—C23—C24—C19	0.8 (8)
S1—C1—C6—C5	−177.9 (4)	C20—C19—C24—C23	−1.1 (7)
C4—C5—C6—C1	1.8 (9)	S2—C19—C24—C23	−178.2 (4)
N1—N2—C7—C8	−178.4 (4)	N4—N5—C25—C26	−175.5 (4)
N2—C7—C8—C9	−6.3 (7)	N5—C25—C26—C27	−15.6 (7)
N2—C7—C8—C13	172.8 (4)	N5—C25—C26—C31	161.8 (4)
C13—C8—C9—C10	−0.7 (7)	C31—C26—C27—C28	−0.1 (7)
C7—C8—C9—C10	178.5 (5)	C25—C26—C27—C28	177.4 (4)
C8—C9—C10—C11	0.4 (8)	C26—C27—C28—C29	−0.7 (7)
C9—C10—C11—C12	−0.3 (7)	C36—N6—C29—C30	−35.3 (7)
C9—C10—C11—N3	−178.7 (5)	C32—N6—C29—C30	−178.2 (5)
C14—N3—C11—C12	142.9 (6)	C36—N6—C29—C28	148.0 (5)
C18—N3—C11—C12	−11.8 (7)	C32—N6—C29—C28	5.1 (7)
C14—N3—C11—C10	−38.8 (8)	C27—C28—C29—C30	0.3 (7)
C18—N3—C11—C10	166.4 (5)	C27—C28—C29—N6	177.3 (4)
C10—C11—C12—C13	0.6 (7)	N6—C29—C30—C31	−176.1 (4)
N3—C11—C12—C13	178.9 (5)	C28—C29—C30—C31	0.9 (7)
C11—C12—C13—C8	−1.0 (8)	C29—C30—C31—C26	−1.7 (8)
C9—C8—C13—C12	1.0 (7)	C27—C26—C31—C30	1.3 (7)
C7—C8—C13—C12	−178.2 (5)	C25—C26—C31—C30	−176.3 (4)
C11—N3—C14—C15	174.4 (7)	C29—N6—C32—C33	−168.9 (5)
C18—N3—C14—C15	−30.4 (10)	C36—N6—C32—C33	46.7 (7)
N3—C14—C15—C16	30.9 (12)	N6—C32—C33—C34	−49.6 (8)
C14—C15—C16—C17	−30.7 (11)	C32—C33—C34—C35	50.7 (8)
C15—C16—C17—C18	32.7 (12)	C33—C34—C35—C36	−50.0 (8)
C14—N3—C18—C17	30.2 (9)	C29—N6—C36—C35	168.0 (5)
C11—N3—C18—C17	−173.8 (6)	C32—N6—C36—C35	−47.1 (8)
C16—C17—C18—N3	−32.9 (11)	C34—C35—C36—N6	49.5 (8)

### (E)-4-Chloro-N'-[4-(piperidin-1-yl)benzylidene]benzenesulfonohydrazide (III) Hydrogen-bond geometry (Å, º)

**Table:**

*D*—H···*A*	*D*—H	H···*A*	*D*···*A*	*D*—H···*A*
N1—H1*N*···O2^i^	0.83 (4)	2.26 (5)	3.025 (5)	153 (5)
N4—H4*N*···O4^ii^	0.84 (5)	2.29 (5)	3.115 (6)	169 (5)

Symmetry codes: (i) *x*, *y*+1, *z*; (ii) *x*, *y*−1, *z*.
